# Role of Inflammatory Cytokines in Rheumatoid Arthritis and Development of Atherosclerosis: A Review

**DOI:** 10.3390/medicina59091550

**Published:** 2023-08-26

**Authors:** Dražen Bedeković, Ivica Bošnjak, Sandra Šarić, Damir Kirner, Srđan Novak

**Affiliations:** 1 Department of Cardiovascular Diseases Internal Medicine Clinic, University Hospital Osijek, J. Huttlera 4, 31000 Osijek, Croatia; dribosnjak79@gmail.com (I.B.); smakarovic36@gmail.com (S.Š.); damir.kirner@gmail.com (D.K.); 2 Faculty of Medicine Osijek, Department of Internal Medicine, Josip Juraj Strossmayer University, J. Huttlera 4, 31000 Osijek, Croatia; 3 Department of Rheumatology and Clinical Immunology, University Hospital Rijeka, Braće Branchetta 20/1, 51000 Rijeka, Croatia; srdan.novak@gmali.com; 4 Faculty of Medicine Rijeka, Department of Internal Medicine, University of Rijeka, Braće Branchetta 20/1, 51000 Rijeka, Croatia

**Keywords:** rheumatoid arthritis, chronic inflammation mechanisms, cardiovascular risk, cardiovascular mortality, medications used in rheumatoid arthritis

## Abstract

Uncontrolled chronic inflammation results in cardiovascular disease and early death. In this review, we studied the impact of rheumatoid arthritis on the cardiovascular system, including the early and accelerated development of atherosclerosis and its clinical manifestations, focusing on the inflammatory mechanisms leading to arterial wall damage, rapid atherosclerotic plaque formation, and thrombosis. Furthermore, the effect of medications used to treat rheumatoid arthritis on the cardiovascular system was studied. The effect of chronic inflammation and medication on traditional cardiovascular risk factors is not the main subject of this review. We observed that uncontrolled chronic inflammation and some medications directly impact all the stages of atherosclerosis. In conclusion, reducing inflammation and maintaining long-term remission in rheumatoid arthritis may prevent early atherosclerosis. We believe that this review will encourage a better interdisciplinary approach to the management of these patients and further research in this field.

## 1. Introduction

Rheumatoid arthritis (RA) is a chronic inflammatory systemic disease that primarily affects the synovial membrane, cartilage, and bones of small- and medium-sized joints, leading to chronic damage and pannus formation; however, blood vessels and various internal organs are also often affected [1,2,3,4,5]. The prevalence of RA in the general population is approximately 1%, and women are predominantly affected. RA results in significant disability, socioeconomic consequences, and a short lifespan of 5 to 18 years, mainly attributable to increased cardiovascular (CV) morbidity and mortality [1,6,7]. The causes of RA remain unknown, but being of female sex and a family history of RA are known risk factors. Known triggers for RA include exposure to bacterial or viral infections, especially bacteria that cause periodontal disease or the Epstein–Barr virus; trauma, bone, or joint fractures; cigarette smoking; and obesity. Typically, the symptoms of RA develop slowly over several weeks or months and can range from mild to severe. They include articular symptoms, such as pain and swelling; morning stiffness or stiffness after prolonged rest lasting >30 min; symmetrical involvement and loss of function; general symptoms such as fatigue, fever, and weight loss; and extra-articular symptoms such as rheumatoid nodules, cardiopulmonary disease, eye disease, Sjogren’s syndrome, rheumatoid vasculitis, neurological manifestations, and Felty’s syndrome [1,2,3,4,5,6,7,8,9,10,11,12,13,14,15,16].

Atherosclerosis and its complications are the most common CV manifestations of RA and are the leading cause of death in patients with RA. Moreover, two major mechanisms of chronic inflammation have a substantial impact on CV risk causing direct damage to the CV system, especially the arteries, and an indirect effect through traditional CV risk factors. These factors synergistically increase CV risk, morbidity, and premature mortality. The traditional risk factors include arterial hypertension, cigarette smoking, dyslipidemia, low levels of physical activity, and diabetes/insulin resistance [17]. Compared with healthy individuals, patients with RA experience adverse effects on the development of CV disease (CVD) due to traditional CV risk factors, including arterial hypertension that increases the risk by 53–73% (in most but not all studies); cigarette smoking increases risk by 25–50%; dyslipidemia increases risk by 73% (difficulty in assessment due to “lipid paradox”); low physical activity is neutral to the increased risk; diabetes/insulin resistance is double; and obesity increases risk by 16% (in most but not all studies) [18,19,20,21,22,23,24,25,26,27,28,29,30,31,32,33,34,35,36,37]. However, the effects of chronic inflammation in RA and of medications used for its treatment on traditional CV risk factors are not the subjects of this article. The focus of this review was to gather all relevant data and knowledge, analyze and discuss the role and mechanisms of chronic inflammation, as well as the effect of medications used for treating RA, on the CV system. We believe that this review will contribute to an improved understanding and encourage further research to prevent early atherosclerosis development and its consequences in patients with RA.

## 2. Materials and Methods

### 2.1. Objective

To systematically gather and analyze all relevant data regarding the direct effects of chronic inflammation associated with RA, as well as the effect of RA medications on the cardiovascular system.

### 2.2. Data Sources

A systematic search and review of the available relevant literature was conducted in the medical databases Web of Science, Scopus, PubMed, and Cochrane Library.

### 2.3. Keywords Used in Article Selection

Rheumatoid arthritis, chronic inflammation mechanisms, cardiovascular risk, cardiovascular mortality, coagulation mechanisms, medications in rheumatoid arthritis, non-steroidal anti-inflammatory drugs (NSAIDs), glucocorticoids/corticosteroids, methotrexate, hydroxychloroquine, leflunomide, cyclosporine, azathioprine, biologic medications, infliximab, etanercept, adalimumab, tocilizumab, abatacept, rituximab, certolizumab, tofacitinib, golimumab, sarilumab, anakinra, canakinumab, baricitinib, and statins.

### 2.4. Article Selection: Inclusion and Exclusion Criteria

Inclusion criteria: Data from meta-analyses, large randomized controlled trials, prospective clinical trials, relevant reviews, as well as data from the European Society of Cardiology and European Alliance of Associations for Rheumatology guidelines, were considered to be the most relevant. Smaller studies were included only if no other data were available or if we considered them crucial. Only two case reports, one expert opinion, and one meta-analysis (critics) were included on specific topics. All the known study limitations are listed in Table S1. The selected and analyzed articles consisted of important data on the following topics: mechanisms of chronic inflammation in RA and its effect on the CV system, cardiovascular disease development in RA, and the effect of medications used to treat RA on the CV system.

Exclusion criteria: Case reports, pilot projects, studies, meta-analyses, and reviews with questionable methodology or reliability; underpowered studies; studies with no full-text available; studies with a population aged ˂18 years; and studies not directly related to the investigation topics were generally excluded.

We selected abstracts of published studies according to the inclusion criteria; if suitable, we analyzed the full text. All important data were extrapolated and copied into pre-prepared tables and were analyzed by at least one cardiologist and rheumatologist. Special attention was paid to the statistical data in articles, which were reviewed by at least one of two employed statisticians. The statistician assessed the size and representativeness of the sample and the use of statistical methods and their adequacy. The final analysis was performed by all the authors. The precise selection process is described in Section 3.1 and in the Supplementary Materials.

### 2.5. Limitations

We accepted all articles written in English or German. Studies involving populations under 18 years of age were excluded from the review. Some studies did not declare the number of participants in a meta-analysis or review but were accepted because of the importance of the topic, or because only a few articles were available on certain topics. Some limitations of the included studies were identified.

### 2.6. Study Design

Review.

### 2.7. Review Period

Studies from 1986 to 2022.

## 3. Results

### 3.1. Article Selection Process

**Figure 1:**
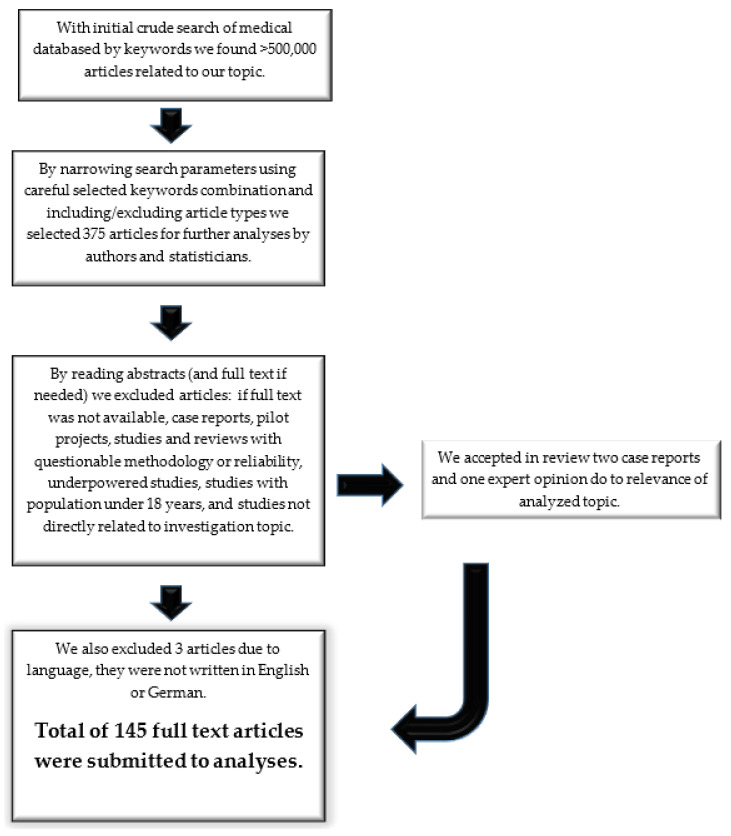
Article selection process for review—schematics.

More than 500,000 published articles were initially crudely screened in databases using the pre-selected keywords. We initially selected 375 abstracts by narrowing the search parameters and carefully combining the main keywords with others (e.g., rheumatoid arthritis and biologic medications) and concurrently included or excluded article types according to the inclusion and exclusion criteria. From 375 abstracts, including 61 meta-analyses, we identified 145 articles that were suitable for this review according to the inclusion and exclusion criteria (Figure 1). We excluded 3 articles that were not in English or German, along with 24 that lacked full-text accessibility, 1 that constituted a letter to the editor, 2 based on the recommendation of a statistician, 2 involving pediatric study population, 2 studies describing obsolete diagnostic procedures, and 194 that were not closely related to our topic or were repetitive. We divided the articles into four categories. The first two categories provided general information about RA and CVD and traditional risk factors, and they served as an introduction, whereas the last two categories were analyzed for the effects of chronic inflammation mechanisms in RA (52 articles) and the effects of medications used in RA (62 articles) on the CV system.

### 3.2. Analysis Results

We analyzed 52 articles for the effect of RA and 62 for the effect of medications on the CV system. Article characteristics are presented in Table S1.

#### 3.2.1. Chronic Inflammation

RA arises from the interplay between genetic susceptibility and environmental triggers. The most important genetic risk is the presence of the DRB1*04:01 gene, a shared epitope that induces the binding of post-translationally modified (citrullinated) proteins, and the PTPN22 gene, which increases citrullination [38]. The major environmental risk factors include tobacco smoking, being of female sex, being of advanced age, and certain foods [38,39,40]. Autoimmune processes include the recognition of synovial tissue self-antigens, such as type II collagen, proteoglycans, and cartilage protein gp39 [41,42,43]. First, the joint intimal lining expands, causing synoviocyte activation and proliferation; they then begin to secrete pro-inflammatory cytokines, such as tumor necrosis factor (TNF), interleukin-1 (IL-1), interleukin-6 (IL-6), metalloproteinases, prostaglandins, and leukotrienes. Synovial invasion into the adjacent articular structures damages the cartilage and bone, manifesting as joint swelling. Second, synovial layer proliferation contributes to the activation of neutrophils and T- and B-lymphocytes, which infiltrate the joints and secrete cytokines and proteinases that further damage the extracellular matrix. Effector CD4+ T cells play a crucial role in disease progression and are characterized by an imbalance between Th1/Th17 and regulatory T cells [44,45]. Atherosclerosis and synovial inflammation in RA share a common pathway, and sustained synovial secretion of inflammatory mediators elicits chronic low-grade activation and dysfunction of the vascular endothelium, thereby expediting the development of atherosclerosis in RA [46].

Citrullinated synovial proteins induce the production of RA-specific autoantibodies (anti-CCP) [47,48], which can increase the risk of ischemic heart disease (6.5% vs. 2.6%, odds ratio [OR]: 2.58, 95% confidence interval [CI]: 1.17–5.65) [49]. Anti-CCP antibodies are associated with early subclinical atherosclerosis and promote atherosclerotic plaque formation by targeting citrullinated sarcomeric proteins, fibrinogen, and vimentin [50,51,52]. Several studies addressing anti-CCP positivity reported that it is associated with higher total mortality and an increase in fatal CV outcomes but not with heart failure or recurrent ischemia [52,53,54,55]; however, large studies did not confirm this finding [56,57]. Other antibodies are also possibly associated with CVD risk, such as antibodies against carbamylated proteins (anti-CarP) and malondialdehyde–acetaldehyde adducts [53]. The combination of genetic and environmental triggers also leads to constant activation and clonal expansion of specific CD4 + CD28 null T cell subsets, and especially the loss of CD28, a co-stimulatory molecule required for normal T cell activation, which correlates with seropositivity and extra-articular RA manifestations [58]. The possible direct cytotoxic effects of these cells on endothelial cells, along with their induced dysfunction, can cause early atherosclerosis and its complications [59,60,61]. This strongly stimulates the activity and recruitment of macrophages and T cells to the plaque, contributing to reactive oxygen species production, inhibiting collagen production, stimulating matrix metalloproteinases, and inducing tissue factor expression that is an independent predictor of future acute coronary events in patients with RA (OR: 3.01, 95% CI: 1.1–8.25, *p* = 0.023) [62,63]. The activated endothelium promotes the binding of neutrophils, monocytes, and platelets, which is further potentiated with neutrophils, IL-8, and monocyte CCL2 chemokines. Adherent neutrophils and monocytes promote further activation of the vascular endothelium with PAR-1. Neutrophils exposed to activated platelets form intravascular neutrophil extracellular traps, which by the expression of endothelium-activating proteases, histones, and tissue factors, promotes the creation of intravascular pro-inflammatory and prothrombotic milieus [14].

C-reactive protein (CRP) and fibrinogen are less likely to be causally associated with atherogenesis according to newer studies; however, pro-inflammatory cytokines, interleukin-6 (IL-6), interleukin-18 (IL-18), and tumor necrosis factor-α (TNF-α) could be directly etiologically associated with atherogenesis through the regulation of inflammatory cascades [64,65]. One prospective study, and a meta-analysis of 29 studies investigating six pro-inflammatory cytokines (IL-6, IL-18, matrix metalloproteinase-9 [MMP-9], soluble CD40 ligand [sCD40L], and TNF-α) in coronary heart disease, concluded that higher baseline levels of IL-6, IL-18, and TNF-α were associated with a 10–25% higher risk of non-fatal myocardial infarction and CV death [66].

Chronic inflammation also has pro-coagulant and pro-oxidant effects mediated using several mechanisms, including increased expression of adhesion molecules for tissue factors, reduced synthesis of nitrogen oxide and thrombomodulin, and induction of nicotinamide adenine dinucleotide phosphate (NADPH) oxidases, causing further endothelium dysfunction [67,68]. Significantly increased levels of tissue factors, fibrinogen, von Willebrand factor, factor (F) VIII, activated FXIIa, and markers of thrombin synthesis have been observed in patients with RA with high inflammatory activity [67,69]. Activated platelets are a crucial element in the development of acute CV syndromes, as well as in atherosclerotic plaque formation, and elevated platelet counts could serve to assess RA activity [70,71]. Collectively, these mechanisms shift the hemostatic balance towards a prothrombotic state in RA [52]. CV risk estimation in the general population is based on different risk scores that underestimate CV risk in patients with RA. This phenomenon is believed to be primarily driven by chronic inflammation, and it has been observed in various studies, such as the HOOM and CARRÉ studies, thereby affecting risk assessment models such as the Framingham score or SCORE system [72].

IL-6 and TNF-α are independently associated with a higher coronary calcium score and increased CV risk [52,73,74]. Cytokine influence begins very early in RA, mostly affecting the carotid and coronary arteries, and it is associated with a significant proportion of acute CV events [46]. High-grade inflammation is associated with increased CV morbidity and mortality, with the CRP level and erythrocyte sedimentation rate (ESR) being independent markers [57,75,76,77]. The use of CRP, or highly sensitive CRP, as a predictor of CV risk in modified CV risk calculators has not been adopted in standard cardiology practice [78].

Endothelial dysfunction and signs of atherosclerosis present very early in RA and is the result of complex interactions among modifiable CV risk factors, genetic predisposition, chronic inflammation, pro-oxidative stress, prothrombotic status, and metabolic abnormalities (insulin resistance and dyslipidemia) [78,79,80,81]. According to Gonzalez-Gay et al., endothelial dysfunction is worsened by long-standing RA of >20 years compared with RA of <7 years; however, the success of inflammation control has not been investigated [81]. Recently, critical limb ischemia was reported in a 27-year-old man with psoriasis who presented without any CV risk factors [82]. Endothelial dysfunction in RA can be assessed measuring circulating soluble adhesive molecules, such as E-selectin, P-selectin, intracellular adhesion molecule-1 (ICAM-1), vascular cell type 1 adhesion molecule (VCAM-1), and flow-mediated arterial dilatation, all of which are suggested for use in CV risk assessment; these methodologies are supported by a meta-analysis involving 20 studies including 852 patients with RA [83].

Duplex atherosclerosis screening is a widely used method for the detection of atherosclerotic plaques that are predictive of CV disease [83]. Assessing CV burden in RA by measuring carotid intima–media thickness is no longer recommended [17,27]; however, detection of carotid plaque formation has a predictive value, with a pronounced effect in early RA and among male patients with a higher inflammatory burden [17,84].

Flow-mediated dilatation, augmentation index, pulse wave velocity, coronary artery calcification score (CAC), SPECT/CT, PET/CT, and PET/MRI are also used to assess atherosclerotic burden; however, non-imaging methods have many limitations and confounding factors [36]. CAC, a measure of coronary artery calcification and subclinical atherosclerosis, is closely related to the degree of atherosclerotic plaque burden and is a strong predictor of CV events [36,85]. Coronary artery calcification was independently associated with older age and hypertension, whereas abdominal aorta calcification was independently associated with older age and erosive arthritis [85].

#### 3.2.2. Influence of Medications

##### NSAIDs

Non-steroidal anti-inflammatory drugs (NSAIDs) and cyclooxygenase-2 inhibitors (COX2 inhibitors) have good anti-inflammatory and analgesic effects, but they increase the risk of acute CV diseases, particularly stroke and myocardial infarction; this occurs particularly with diclofenac and rofecoxib [32,86,87,88,89,90,91,92]. Other adverse effects, such as an increased risk of atrial fibrillation and heart failure, can induce or aggravate arterial hypertension, acute or chronic kidney damage, and gastrointestinal complications, especially in older patients with multiple comorbidities [90,91,92,93,94]. Inhibition of different isoenzymes of cyclooxygenase (COX) and a decrease in prostaglandins at inflammatory sites increase thromboxane A2 (COX-1) and decrease prostaglandin I2 (COX-2) production, which may lead to vasoconstriction, platelet activation, hypertension, accelerated atherosclerosis, renal sodium retention with peripheral edema and heart failure, and increased CV morbidity and mortality [88,92,94,95,96]. The Vioxx Gastrointestinal Outcomes Research (VIGOR) and the Adenomatous Polyp Prevention on Vioxx (APPROVe) trials led to rofecoxib withdrawal due to the high risk of thrombotic events. This was supported by a meta-analysis involving 28 RA studies that reported an 18% increased risk of all CV events (RR, 1.18; 95% CI 1.01 to 1.38; *p* = 0.04) and strokes with a greater effect with COX-2 inhibitors (RR, 1.36; 95% CI 1.10 to 1.67; *p* = 0.004) than that with nonselective NSAIDs (RR, 1.08; 95% CI 0.94 to 1.24; *p* = 0.28) [88,89,91]. An analysis of 19 studies including patients with RA and osteoarthritis revealed a significantly increased risk of CV events with diclofenac and rofecoxib and a non-significant increased risk with celecoxib [97]. NSAIDs should be prescribed at the lowest effective doses and for the shortest possible duration [72,91,93]. Gastric prophylaxis is also recommended, especially if NSAIDs are combined with glucocorticoids, in older adults and in patients with a moderate to high risk of peptic ulcer disease [93]. The use of acetylsalicylic acid for the primary prevention of CV disease in patients with RA is not recommended [17,78].

##### Glucocorticoids

Glucocorticoids have potent anti-inflammatory and immunosuppressive effects and are widely used in RA treatment. The addition of low-dose glucocorticoids (below 7.5 mg/daily prednisone) to disease-modifying antirheumatics (DMARDs) in early RA slows the radiological progression of bone destruction [92,98]. Long-term use of glucocorticoids at high and low doses significantly increases CV risk, with an unfavorable impact on lipid metabolism, obesity, insulin production, insulin resistance, and blood pressure [22,36,99]. Numerous studies have found an increased incidence of all-cause and CV mortality, hypertension, hyperglycemia, diabetes, osteoporosis, and myocardial infarction with dose- and time-dependent glucocorticoid usage [22,88,92,100]. According to current guidelines, glucocorticoids should be used at the lowest possible dose, continuation should be regularly reassessed, and remission withdrawal should be considered [101,102].

##### Classical DMARDs

DMARDs, especially methotrexate (MTX), sulfasalazine, and hydroxychloroquine, have beneficial effects on CV risk [36,83,100,103,104,105]. MTX is an antifolate immunosuppressive drug that inhibits neutrophil chemotaxis and synthesis of pro-inflammatory cytokines, as well as exerts antiatherogenic and cardioprotective effects [102,105]. MTX was associated with a 21% lower overall risk of CV events (95% CI 0.73–0.87, *p* < 0.001) and an 18% lower risk of myocardial infarction (95% CI: 0.71–0.96, *p* = 0.01) [103]. A study on CAC, using computed tomography, reported a lower coronary calcification burden with MTX use in patients with RA [85]. Another meta-analysis including 28 studies reported an overall 21% CV risk reduction with MTX (RR, 0.72; 95% CI 0.57 to 0.91; *p* = 0.007) as well as an 18% risk reduction in myocardial infarction—a trend towards a decreasing risk of heart failure, whereas it revealed no effect on strokes and major adverse cardiac events [88]. An older meta-analysis including 18 studies also reported a similar reduced risk of CV events in patients with RA treated with MTX [106,107]. Several studies have reported that MTX increases total cholesterol, LDL, HDL, and triglyceride levels in RA, with a possible explanation suggesting that it reflects the normalization of lipid levels due to the suppression of inflammation, without increasing CV risk [74]. Hydroxychloroquine exerts antirheumatic effects by targeting autoantigen processing in macrophages, suppressing T-lymphocytes, and neutralizing the prothrombotic effects of antiphospholipid antibodies [91,104]. Hydroxychloroquine improves lipid and glycemic indices and reduces the risk of thromboembolic events and CV risk [72,91,104,108,109]. Several reports have asserted the consideration of hydroxychloroquine cardiotoxicity (restrictive cardiomyopathy and conduction disorders). However, in those cases, patients had prolonged use of large cumulative doses; notably, the use of hydroxychloroquine is considered safe at therapeutic doses with periodic ECG monitoring [104]. Combination therapy with MTX, sulfasalazine, and hydroxychloroquine also decreases CV risk by improving the reduction in inflammation, increase in HDL, and decrease in LDL and by enhancing the ratio of total cholesterol to HDL. Notably, this combination therapy has shown superiority over MTX monotherapy or a combination of MTX and etanercept; however, the study only included patients with early RA with high disease activity who were naïve to DMARDs [110]. Azathioprine, cyclosporine, and leflunomide increase the risk of CV events by 80% that of MTX monotherapy [111,112]. Leflunomide has potent anti-inflammatory and immunomodulatory effects; however, it increases the risk of hypertension, which has been reported in 2.1–10.6% of cases in different studies [91,113]. Cyclosporine is used for the treatment of severe early RA and has many adverse effects; careful monitoring is advised when using cyclosporine [91,113]. Azathioprine is a purine analog with rare adverse effects, which include angina, renal and subclavian vein thrombosis, hypotension, and cardiogenic shock [91,113].

##### Biologic Agents

Biologics target inflammatory cytokines (TNF-α and IL-6) and cytokine receptors, interrupting the vicious cycle of inflammation, and are recommended even in early RA with low-severity inflammatory arthritis [91,112]. By suppressing inflammation and maintaining low disease activity, they significantly reduce the risk of CV and incidence of myocardial infarction, heart failure, and cerebrovascular events in patients with RA [91,113,114,115,116,117]. Anti-TNF agents neutralize soluble- and/or membrane-bound TNF and act as monoclonal antibodies or soluble receptors [91]. In addition to the anti-inflammatory effect of anti-TNF-α therapy, and the consequential improvement in joint function, they may indirectly lead to increased levels of physical activity, which will subsequently decrease the incidence of other CV risk factors, such as diabetes mellitus and hypertension [114]. Karpouzas et al. reported slower non-calcified coronary plaque progression with longer usage of biologics, independent of inflammation, prednisone dose, and statin use [118].

A recently published meta-analysis, including 26 longitudinal studies addressing the question of anti-TNF therapy’s effect on body mass index (BMI), found a small increase in body weight and BMI—on average 0.90 kg, 2.34 kg, and 2.27 kg for infliximab, etanercept, and adalimumab, respectively, at 4 and 104 weeks of follow-up [119]. The ATTACH study reported that anti-TNF therapy increased mortality or worsened heart failure in patients with moderate to severe chronic heart failure, especially those with an ischemic etiology, but the RENAISSANCE and RECOVER clinical trials did not confirm this for etanercept [120]. A possible reduction in insulin resistance with anti-TNF therapy was reported in a meta-analysis of 12 studies [121]. A meta-analysis of studies considering the impact of biologics (tocilizumab, abatacept, rituximab, and TNF inhibitors) on CV risk and safety reported fewer CV events with rituximab [122]. A large meta-analysis, which included 43 biological registers and 27 publications, addressed the issue of biologics’ safety and effect on mortality. It reported that overall mortality and CV events were significantly reduced in patients treated with anti-TNFs with relative risk (RR) = 0.60 [95% CI 0.38–0.94] and RR = 0.62 [95% CI 0.44–0.88], respectively, with no effect on the risk of neoplasm but a significant increase in infections during anti-TNF treatment (RR = 1.48 [1.18–1.85]) compared to those who were treated with classical DMARDs [123]. Another meta-analysis including 28 studies of patients with RA reported that anti-TNF treatment was significantly associated with an overall CV risk reduction of approximately 30% [88]. Cheung et al. analyzed the effect of anti-TNF therapy on subclinical atherosclerosis, and six studies measured at least one parameter before and after treatment (24th and 52nd weeks), which included intima–media thickness, pulse wave velocity, and an augmentation index; they observed that anti-TNF therapy had no effect on all three parameters at the 24th week and on intima–media thickness at the 52nd week [124]. An older meta-analysis including 32 studies by Daien et al. demonstrated increased total cholesterol and HDL levels and unchanged LDL levels with long-term anti-TNF therapy, with uncertain effects on CV risk [125]; however, the total cholesterol/HDL ratio was not significantly altered with anti-TNF therapy [74]. A meta-analysis by Zhao et al. reported an increased incidence of hypertension associated with some anti-TNF therapies (OR = 1.8896, 95% CI: 1.35–2.65), as well as an increased incidence of hypertension with longer therapy durations, associated with certolizumab but not with etanercept, tofacitinib, infliximab, and golimumab [126]. Results from a new Korean observational study revealed no increase in the incidence of hypertension with biologics than that with classical DMARDs, in general [127]. A neutral effect of anti-TNF agents on HA incidence was reported by Desai et al. from a large cohort study [128].

The effect of non-anti-TNF agents—abatacept, tocilizumab, sarilumab, anakinra, and rituximab—on CV risk has also been evaluated; abatacept was found to have a 20% greater CV risk reduction than that of anti-TNF agents; tocilizumab had the same effect on CV risk as MTX monotherapy; anakinra showed improved vascular and left ventricular function in a small placebo-controlled study; sarilumab and rituximab had a neutral effect on CV events; and canakinumab, an IL-1 inhibitor administered at 150 mg for every 3 months had a significantly lower rate of myocardial infarction compared with that of the placebo [76,129,130,131,132,133,134]. Several studies have consistently reported that tocilizumab was associated with increased total cholesterol, HDL, LDL, and triglyceride levels, [74,91]. But the MEASURE study demonstrated that the concentration of proatherogenic small and dense LDL particles was not increased [135]. No studies have reported an increase in CV events with tocilizumab [91].

##### Small Molecule Inhibitors of Janus Kinase

Tofacitinib and baricitinib, newer small-molecule inhibitors of janus kinase (JAK), increase total and LDL cholesterol levels by 10–20% [136]. Increased dose-dependent LDL and HDL levels were reported with baricitinib use; the mean change was 13.15 mg/dL (95% CI: 8.89–17.42) and 5.40 mg/dL (95% CI: 3.07–7.74), respectively [130]. A recently published pooled cohort study of 3492 patients with RA, with more than 7860 patient-years of exposure to baricitinib, did not reveal a significant association between baricitinib treatment and the occurrence of CV events or congestive heart failure [137]. A meta-analysis including 20 studies investigated the influence of biologic agents and tofacitinib on the lipid profile of patients with RA and revealed increased cholesterol (OR 4.64; 95% CI 2.71, 7.95, *p* < 0.001), HDL (OR 2.25; 95% CI 1.14, 4.44, *p* = 0.020), and LDL (OR 4.80; 95% CI 3.27, 7.05, *p* < 0.001) levels; however, despite this effect on lipid levels, better inflammation control with those medications appears to result in lower mortality and reduced incidence of CV events [138].

With the exception of the ENTRACTE trial, which compared tocilizumab and etanercept, no head-to-head study has been conducted on the impact of biological agents on CV risk and safety. The ENTRACTE trial reported no significant difference in CV events between the tocilizumab and etanercept groups [85]. Observational studies have reported a higher incidence of myocardial infarction among older patients with anti-TNF inhibitors than with abatacept and tocilizumab, and no difference in CV risk was observed when comparing tocilizumab with abatacept [76,139].

##### Statins

The risk of RA may be lower in patients with a longer duration or high intensity of statin use [139]. Treatment with statins is beneficial in lowering CV risk in patients with RA due to their lipid-lowering and other pleiotropic effects that slow down coronary non-calcified plaque progression and suppress the effects of inflammation on plaque progression and CAC [78,118,140,141,142,143].

## 4. Discussion

### 4.1. Chronic Inflammation

RA has a complex etiology that involves a combination of genetic susceptibility and environmental triggers. The most prominent genetic risk is the presence of the human leukocyte antigen DRB1*04:01 gene, which encodes a shared epitope—a 5-amino acid sequence—inducing the binding of post-translationally modified (citrullinated) proteins. Another genetic risk factor is PTPN22, which increases citrullination [38]. The major environmental risk factors include tobacco smoking, being of female sex, being of advanced age, and certain foods [38,39,40]. Autoimmune processes include the recognition of synovial tissue self-antigens, such as type II collagen, proteoglycans, and cartilage protein gp39 [41,42,43]. The activation and combination of these two major mechanisms are crucial for joint destruction and bone erosion in RA. Initially, the joint’s intimal lining expands, causing synoviocyte activation and proliferation. These cells start releasing pro-inflammatory cytokines (such as TNF-α, IL-1, and IL-6), metalloproteinases, prostaglandins, and leukotrienes. Synovial invasion into the adjacent articular structures damages the cartilage and bone, manifesting as joint swelling. Subsequently, synovial layer proliferation contributes to the activation of neutrophils and T- and B-lymphocytes, leading to their infiltration into the joints. This infiltration results in the secretion of cytokines and proteinases that further damage the extracellular matrix. Effector CD4+ T cells play a crucial role in disease progression and are characterized by an imbalance between Th1/Th17 and regulatory T cells [44,45]. As the pathogenic processes of atherosclerosis and synovial inflammation in RA share a common pathway, the understanding of these processes represents a cornerstone of CV risk management in patients with RA. Sustained synovial secretion of inflammatory mediators leads to chronic low-grade activation and dysfunction of the vascular endothelium, promoting the accelerated development of atherosclerosis in RA [46].

Citrullinated synovial proteins induce the production of anti-CCP autoantibodies, largely linked to RA. These antibodies are associated with more severe forms of RA [47,48] and have been extensively studied in the context of CV. According to López-Longo et al., anti-CCP antibody titer levels > 25 units/mL carry a higher risk of ischemic heart disease (6.5 vs. 2.6%, OR: 2.58, 95% CI: 1.17–5.65) without affecting mortality [49]. Previous studies clarified the association of citrullinated proteins and anti-CCP antibodies with early subclinical atherosclerosis and atherosclerotic plaque promotion. This association involves the interaction of anti-CCP antibodies with citrullinated fibrinogen in plaques, inducing inflammation and contributing to heart failure. Additionally, anti-CCP antibodies targeting citrullinated sarcomeric proteins, namely fibrinogen and vimentin, can lead to heart issues independently of coronary artery disease [50,51,52]. However, these studies had important limitations, such as the inability to demonstrate a direct anti-CCP complex in plaque [50] and many false-positive fluorodeoxyglucose uptake results [51]. Several studies addressing anti-CCP positivity reported that it was associated with higher total mortality and increased fatal CV outcomes but not with heart failure or recurrent ischemia [52,53,54,55]; study limitations are listed in Table S1. Conversely, other large studies did not find a significant association between anti-CCP and rheumatoid factor positivity and CV morbidity and mortality, [56,57], which we consider to have greater relevance. Moreover, other antibodies present in RA are possibly associated with CVD risk, including antibodies against anti-CarP and malondialdehyde–acetaldehyde adducts [53]. Genetic susceptibility and environmental triggers also lead to constant activation and clonal expansion of specific CD4 + CD28 null T cell subsets, which play a crucial role in the pathogenesis of RA. The loss of CD28, a co-stimulatory molecule required for normal T cell activation, correlates with seropositivity and extra-articular RA manifestations [58]. Increased expression of perforin and killer cell immunoglobulin-like receptors in these cells, with potential direct cytotoxic effects on endothelial cells and their dysfunction, can cause early atherosclerosis, plaque rupture, and thrombosis [59,60,61]. This expression strongly stimulates the activity and recruitment of macrophages and T cells to the plaque, contributing to reactive oxygen species production, inhibiting collagen production, stimulating matrix metalloproteinases, and inducing tissue factor expression [62]. According to Liuzzo et al. [63], the level of CD4 + CD28 null T-cells in patients’ blood was an independent predictor of future acute coronary events in patients with RA (OR: 3.01, 95% CI: 1.1–8.25, *p* = 0.023). The activated endothelium promotes the binding of neutrophils, monocytes, and platelets, which is further potentiated by neutrophils, IL-8, and monocyte CCL2 chemokines. Adherent neutrophils and monocytes promote further activation of the vascular endothelium with PAR-1, creating a vicious cycle that leads to endothelial dysfunction. Neutrophils exposed to activated platelets form intravascular neutrophil extracellular traps, which—by expression of endothelium-activating proteases, histones, and tissue factors—promote the development of intravascular pro-inflammatory and pro-thrombotic milieus [14]. This finding is substantiated by numerous published studies and reviews.

Many studies have focused on the role of inflammatory cytokines in atherogenesis and CV disease development, as well as their use for risk stratification. Newer studies reported that CRP and fibrinogen are less likely to be causally associated with atherogenesis, but pro-inflammatory cytokines (IL-6, IL-18, and TNF-α) could be directly etiologically associated with atherogenesis by regulation of inflammatory cascades [64,65]. In a prospective study with 1514 participants and a meta-analysis of 29 studies with approximately 17,000 participants, Kaptoge et al. [66] studied the roles of five pro-inflammatory cytokines, IL-6 and IL-18, MMP-9, sCD40L, and TNF-α in coronary heart disease and concluded that higher baseline levels of IL-6, IL-18, and TNF-α were associated with a 10–25% higher risk of non-fatal myocardial infarction and CV death, whereas sCD40L and MMP-9 did not show any such association.

Chronic inflammation also has pro-coagulant effects mediated by several mechanisms, including increased expression of adhesion molecules for tissue factors, reduced synthesis of nitrogen oxide and thrombomodulin, and increased pro-coagulant properties of the endothelium [67]. Endothelial dysfunction is further mediated by the induction of NADPH oxidases and dysfunction of antioxidant systems [68]. Significantly increased levels of tissue factors, fibrinogen, von Willebrand factor, factor (F) VIII, activated FXIIa, and markers of thrombin synthesis have been observed in patients with RA with high inflammatory activity [67,69]. Platelets activated by cytokine-sensitized endothelial neutrophils or monocytes or by anti-CCP antibody exposure are key elements in the development of acute CV syndromes and in atherosclerotic plaque formation, recruiting leukocytes to the sites of endothelial damage and inflammation, activating complement and other inflammatory receptors, and releasing cytokines and chemokines [70]. Together, these mechanisms shift the hemostatic balance to a prothrombotic state in RA [52]. A meta-analysis by Zhou et al. [71]. confirmed that platelet counts are elevated in patients with RA and could serve to assess disease activity. CV risk estimation in the general population is based on different risks that underestimate the CV risk in patients with RA. It is hypothesized that chronic inflammation is the key determinant contributing to these underestimations. This is further supported by studies like the HOOM and CARRÉ studies, which are in line with the Framingham score or SCORE system, as suggested by several studies [72]. Nonetheless, these studies have limitations in terms of the methods used to estimate CV risk.

IL-6 and TNF-α are independently associated with a higher coronary calcium score and increased CV risk, favoring the hypothesis that RA-related increased CV risk is associated with higher levels of inflammatory cytokines and their deleterious effects on endothelial cells [52,73,74]. This finding is supported by several published studies. The effect manifests at a very early stage in RA, mostly targeting the carotid and coronary arteries, and is associated with a significant proportion of acute CV events [46]. High-grade inflammation associated with increased CV morbidity and mortality, with CRP levels and ESR as independent markers, was reported in a population-based study spanning a 20-year follow-up period, which is in concordance with several earlier research findings [57,75,76,77]. The use of CRP, or highly sensitive CRP, as a predictor of CV risk in modified CV risk calculators was not adopted in standard cardiology practice [78].

Endothelial dysfunction in RA is the result of complex interactions among modifiable CV risk factors, genetic predisposition, chronic inflammation, pro-oxidative stress, prothrombotic status, and metabolic abnormalities (insulin resistance and dyslipidemia) [78,79]. It is present in a very early stage of RA, even before or within one year of the clinical onset of RA—as well as in arterial wall atherosclerosis—with an increased risk of coronary heart disease and myocardial infarction [80,81]. According to Gonzalez-Gay et al. [81], endothelial dysfunction is worsened by long-standing RA of >20 years compared with RA of <7 years; however, the success of inflammation control was not investigated. A recently described case of critical limb ischemia in a 27-year-old man with an 8-year history of psoriasis (a chronic inflammatory disease similar to RA), and without any CV risk factors, points to inflammation as the main cause of endothelial dysfunction and vascular damage, regardless of the presence of traditional or inherited CV risk factors. However, notably, this is a single case, and the outcomes of inflammation management and articular involvement were not described [82]. Endothelial dysfunction in RA can be assessed by measuring circulating soluble adhesive molecules such as E-selectin, P-selectin, ICAM-1, VCAM-1, and flow-mediated arterial dilatation, all of which are suggested for use in CV risk assessment; this observation is supported by a meta-analysis of 20 studies including 852 patients with RA [83].

Duplex atherosclerosis screening is mostly used for detecting atherosclerotic plaques that are predictive of CV disease [83]. Although carotid intima–media thickness measurements were used in previous investigations to assess CV burden in RA, they are no longer recommended as per ESC guidelines [17,27]. However, the detection of carotid plaque formation has a predictive value with a pronounced effect in early-stage RA and in men with a higher inflammation burden [17,84].

**Figure 2:**
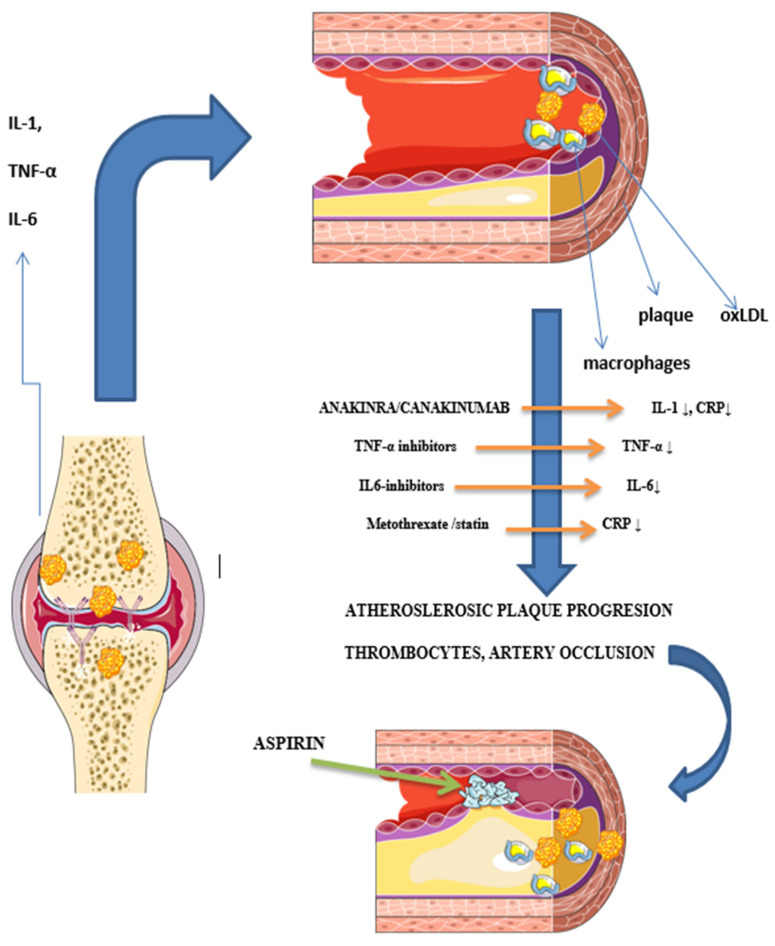
Role of proinflammatory cytokines and medication in vascular damage.

Flow-mediated dilatation, augmentation index, pulse wave velocity, CAC score, SPECT/CT, PET/CT, and PET/MRI are also used to assess atherosclerotic burden; however, non-imaging methods have many limitations and confounding factors [36]. The CAC score, a measure of coronary artery calcification and subclinical atherosclerosis, is closely related to the degree of atherosclerotic plaque burden and is a strong predictor of CV events [36]. Paccou et al. [85] conducted a comparison between asymptomatic patients with RA and healthy controls and reported higher prevalence and severity of both coronary artery calcification and abdominal aorta calcification in patients with RA. CAC was independently associated with older age and hypertension, whereas abdominal aorta calcification was independently associated with older age and erosive arthritis. However, there are some reservations concerning this method; for example, non-calcified plaques cannot be detected. Accelerated atherosclerosis in RA, in addition to epicardial artery disease, can cause microvascular dysfunction. This dysfunction plays a crucial role in regulating myocardial perfusion and contributes to the accelerated development of CV disease (Figure 2).

Proinflammatory cytokines interleukin 1 (IL-1), tumour necrosis factor alpha (TNF-α) and interleukin 6 (IL-6) are interconnected in signalling pathways and they are targets for drugs that are used to treat rheumatic diseases and have gained interest as potential drugs for secondary prevention of ASCVD in RA. ASCVD and rheumatoid synovitis both develop via similar inflammatory mechanisms. The first stage involves endothelial dysfunction, whith inflammatory cells infiltration in the joint capsule and the beginning of plaque formation in the artery’s sub-intima. The decreased anti-oxidative activity in RA patients also encourages LDL oxidation and foam cell production.. *Methotrexate:* Enhances macrophage cholesterol efflux and prevents foams cell differentiation and activation. Upregulates free radical scavenging; improves endothelial function. *TNF-α inhibitors:* TNF-α promotes numerous inflammatory responses associated with atherosclerosis, including induction of vascular adhesion and monocyte/macrophage proliferation. TNF-α impacts lipid metabolism by stimulating liver triglyceride production. Atherosclerosis development and RA inflammation are both slowed down by inhibiting these processes. *Tocilizumab (IL-6):* Decreases inflammatory proteins such as serum amyloid A, and restores the anti-atherogenic function of HDL by increasing HDL cholesterol efflux capacity. *Canakinumab IL-1β:* significantly reduced hsC-reactive protein levels from baseline, as compared with placebo, without reducing the LDL cholesterol level. *Statins:* decrease LDL cholesterol by inhibing HMG-CoA and lowering hsCRP.

### 4.2. Influence of Medications

#### 4.2.1. NSAIDs

NSAIDs and COX2 inhibitors have good anti-inflammatory and analgesic effects; they can paradoxically increase the risk of acute CV diseases, especially stroke and myocardial infarction. In particular, NSAIDs such as diclofenac and rofecoxib inhibit prostacyclin synthesis [32,85,86,87,88,89,90,91,92]. This is supported by strong evidence, as in the study by Gargiulo et al. [90]. Other adverse effects include an increased risk of atrial fibrillation and heart failure, induction, or aggravation of arterial hypertension (due to their effect on sodium and water retention), as well as induction of acute or chronic kidney damage and gastrointestinal complications—especially in elderly patients with multiple comorbidities [90,91,92,93,94]. Inhibition of different isoenzymes of COX and a decrease in prostaglandins at the inflammatory sites increase thromboxane A2 (COX-1) and decrease prostaglandin I2 (COX-2) production, which may lead to vasoconstriction, platelet activation, hypertension, accelerated atherosclerosis, renal sodium retention with peripheral edema, and heart failure [92]. Many trials and meta-analyses supported those conclusions: COX-2 inhibitors increased the risk of CV events by 42% [88,94], highly increased the risk of CV-related mortality (adjusted OR 0.54, 95% CI 0.34–0.86) [94], increased the risk of first-time myocardial infarction within 180 days of initiation of NSAIDs [95], and were associated with a higher relative risk of myocardial infarction with diclofenac and rofecoxib [96]. The VIGOR and APPROVe trials led to rofecoxib withdrawal due to the high risk of thrombotic events, which was supported by a meta-analysis of 28 RA studies published by Roubille et al. This study reported an 18% increased risk of all CV events (RR, 1.18; 95% CI 1.01 to 1.38; *p* = 0.04) and strokes with a greater effect with COX-2 inhibitors (RR, 1.36; 95% CI 1.10 to 1.67; *p* = 0.004) than with nonselective NSAIDs (RR, 1.08; 95% CI 0.94 to 1.24; *p* = 0.28) [88,91]. The highest increase in CV events occurred with rofecoxib (RR, 1.58; 95% CI 1.24 to 2.00; *p* < 0.001), which led to rofecoxib withdrawal, but other NSAIDs did not show significant effects on the risk of myocardial infarction, heart failure, or major adverse cardiac events; however, very few events were included in the analysis and did not provide strong evidence [89]. An analysis of 19 studies with patients with RA and osteoarthritis reported a significantly increased risk of CV events with diclofenac and rofecoxib and a non-significant increased risk with celecoxib. Etoricoxib and rofecoxib significantly increased the risk of hypertension, as did naproxen for stroke; however, not all NSAIDs were included in the investigation and analyzed for cardiovascular side effects [89]. NSAIDs should be prescribed at the lowest effective doses and for the shortest possible duration with caution when prescribing to patients with CV disease or in the presence of CV risk factors. They should be avoided in patients with treatment-resistant hypertension, high CV risk, and severe chronic kidney disease; naproxen or celecoxib are the preferred choices in these diseases [72,91,93]. For patients with pre-existing hypertension, who are on renin-angiotensin system blockers, dose increases or additions of a different drug class should be considered. Gastric prophylaxis is also recommended, especially if NSAIDs are combined with glucocorticoids in the elderly and in patients with a moderate to high risk of peptic ulcer disease [93]. The use of acetylsalicylic acid for the primary prevention of CV disease in patients with RA is not recommended [17,78].

#### 4.2.2. Glucocorticoids

Glucocorticoids have potent anti-inflammatory and immunosuppressive effects and are widely used in RA treatment. The addition of low-dose glucocorticoids (below 7.5 mg/daily prednisone) to DMARDs in early RA slows the radiological progression of bone destruction [92,98]. Long-term use of glucocorticoids at high and low doses significantly increases CV risk, with an unfavorable impact on lipid metabolism, obesity, insulin production, insulin resistance, and blood pressure [22,36,99]. Although a meta-analysis of six randomized controlled trials with 689 patients with RA reported good glucocorticoid safety profiles, numerous studies have found an increased incidence of all-cause and CV mortality, hypertension, hyperglycemia, diabetes, osteoporosis, and myocardial infarction with dose- and time-dependent glucocorticoid usage [22,92,100]. Many registries and large meta-analyses support the conclusions of the effects of glucocorticoids on CV risk and mortality, with some dose-dependent effects. These include the Scotland National Health Service RA database, the General Practice Research Database, and a meta-analysis of 28 studies by Roubille et al. [88]. An older meta-analysis of 37 studies reported poor associations between low-dose glucocorticoids (<10 mg/day) and CV risk factors, beneficial effects on lipid profiles, an increase in insulin resistance or glycemia, and no effect on blood pressure, with a trend of increasing major CV events, myocardial infarction, stroke, and mortality [100]. According to current guidelines, glucocorticoids should be used at the lowest possible dose, continuation should be regularly reassessed, and remission withdrawal should be considered [101,102].

#### 4.2.3. Classical DMARDs

Considering DMARDs, a meta-analysis of observational and prospective studies reported on the beneficial effects of MTX, sulfasalazine, and hydroxychloroquine on CV risk [36,84,100,103,104,105]. MTX is an antifolate immunosuppressive drug that inhibits neutrophil chemotaxis and synthesis of proinflammatory cytokines and exerts antiatherogenic and cardioprotective effects [102,103,104,105]. According to an analysis of 8 prospective studies, 2 retrospective studies, and 694 publications, with 66,334 participants and 6235 events, MTX was associated with a 21% lower overall risk for CV events (95% CI: 0.73–0.87, *p* < 0.001) and an 18% lower risk for myocardial infarction (95% CI: 0.71–0.96, *p* = 0.01) [103]. A study on CAC, using computed tomography, reported a lower coronary calcification burden with MTX use in patients with RA [85]. Another meta-analysis of 28 studies reported an overall 21% CV risk reduction with MTX (RR, 0.72; 95% CI: 0.57–0.91; *p* = 0.007) as well as an 18% myocardial infarction risk reduction, a trend towards a decreasing risk of heart failure, and no effect on strokes and major adverse cardiac events—although the last may be due to the lower number of events resulting in insufficient statistical power to detect a significant effect [88]. An older meta-analysis of 18 studies also reported a reduced risk of CV events in patients with RA treated with MTX: a reduction in overall mortality of 41% and 70% in two studies; a reduction in CV-related morbidity of 89%, 35%, 17%, and 15% in four studies; an 18% risk reduction for myocardial infarction in one study (with a selection bias due to the exclusion of fatal events); and a trend towards risk reduction in three studies. One study showed a 20% reduction in the risk of hospitalization with congestive heart failure (significant bias), and another study showed an 11% reduction in the risk of stroke and a trend towards it [106,107]. Several studies have reported that MTX increases total cholesterol, LDL, HDL, and triglyceride levels in RA, with a possible explanation that it reflects the normalization of lipid levels due to the suppression of inflammation, without increasing CV risk [74]. Hydroxychloroquine has antirheumatic effects by targeting autoantigen processing in macrophages, suppressing T-lymphocytes, and neutralizing the prothrombotic effects of antiphospholipid antibodies [91,104]. Hydroxychloroquine improves lipid and glycemic indices, reduces the risk of thromboembolic events, enhances the elasticity of peripheral arteries and systemic vascular resistance, and reduces CV risk [72,91,104,108]. However, considerations of methodology, transparency, and reproducibility that affect the credibility of the conclusions were asserted in the latter meta-analysis [109]. Several case reports have asserted the consideration of hydroxychloroquine cardiotoxicity (restrictive cardiomyopathy and conduction disorders); however, in those cases, patients had prolonged use of large cumulative doses. The use of hydroxychloroquine is considered safe at therapeutic doses with periodic ECG monitoring [104]. Combination therapy with MTX, sulfasalazine, and hydroxychloroquine also decreases CV risk by improving inflammation reduction, increasing HDL, lowering LDL, and improving the ratio of total cholesterol to HDL, as against MTX monotherapy or in combination with etanercept. However, the study only included patients with early RA with high disease activity and who were naïve to DMARDs [110]. In contrast, azathioprine, cyclosporine, and leflunomide increased the risk of CV events by 80% compared to MTX monotherapy [111,112]. Leflunomide has potent anti-inflammatory and immunomodulatory effects but increases the risk of hypertension, which has been reported in 2.1–10.6% of cases in different studies [91,113]. Cyclosporine is used for the treatment of severe early RA and has many adverse effects, including vasoconstriction, thrombosis, and hypertension; blood pressure and renal function monitoring are advised when using cyclosporine [91,113]. Azathioprine is a purine analog with rare adverse effects, which include angina, renal and subclavian vein thrombosis, hypotension, and cardiogenic shock [91,113].

#### 4.2.4. Biologic Agents

Biologics are used to treat many different autoimmune diseases by targeting inflammatory cytokines (TNF-α and IL-6) and cytokine receptors, interrupting the inflammatory vicious cycle, and are recommended even in early RA with low-severity inflammatory arthritis [91,112]. By suppressing inflammation and maintaining low disease activity, they significantly reduce CV risk and the incidences of myocardial infarction, heart failure, and cerebrovascular events in patients with RA [91,113,114,115,116]. Anti-TNF agents neutralize soluble- and/or membrane-bound TNF and act as monoclonal antibodies or soluble receptors [91].

In a large cohort study of 2757 patients with RA treated with infliximab, etanercept, or adalimumab, a significant increase in heart failure was reported in patients with high disease activity and concomitant glucocorticoid or COX inhibitor therapy, while anti-TNF therapy did not significantly contribute to the risk; only sporadic cases of acute coronary syndromes, arrhythmias, and AV block for infliximab were reported [115]. In a large, retrospective study, Solomon et al. reported that anti-TNF-α therapy may be associated with a reduced CV risk compared with classic DMARD therapy. The incidence rates per 100 person-years for the composite cardiovascular end point for classic DMARD and anti-TNF therapy were 3.05 (95% CI, 2.54–3.65) and 2.52 (95% CI, 2.12–2.98), respectively [113]. Jacobsson et al., in a prospective cohort study with 983 participants [116]; Ljung et al., in a prospective cohort study with 6864 patients with RA [117]; and an earlier meta-analysis of 20 studies by Westlake et al. reported similar conclusions [106]. In addition to the anti-inflammatory effect of anti-TNF-α therapy and the consequential improvement of joint function, they may indirectly lead to increased levels of physical activity, which will subsequently decrease the incidence of other cardiovascular risk factors, such as diabetes mellitus and hypertension [114]. Karpouzas et al. reported slower non-calcified coronary plaque progression with longer biologics usage independent of inflammation, prednisone dose, and statin use [118].

A recently published meta-analysis of 26 longitudinal studies addressing anti-TNF therapy influence on BMI found a small increase in body weight and BMI—which was on average 0.90 kg, 2.34 kg, and 2.27 kg for infliximab, etanercept, and adalimumab, respectively at 4 and 104 weeks of follow-up [119]. The ATTACH study reported that the use of anti-TNF therapy increased mortality or worsened heart failure in patients with moderate to severe chronic heart failure, especially those with an ischemic etiology, but the RENAISSANCE and RECOVER clinical trials did not confirm this for etanercept [120]. A possible reduction in insulin resistance with anti-TNF therapy was reported in a meta-analysis of 12 studies; however, the heterogeneity of the studies was high [121]. A meta-analysis of studies considering the impact of biologics (tocilizumab, abatacept, rituximab, and TNF inhibitors) on CV risk and safety reported fewer CV events with rituximab and neutral effects of others compared to classic DMARDs, but significant heterogeneity on CV outcomes was reported [122]. A large meta-analysis, which included 43 biological registers and 27 publications, addressed the issue of biologics safety and effect on mortality, and it reported that overall mortality and CV events were significantly reduced in patients treated with anti-TNFs: RR = 0.60 [95% CI 0.38–0.94] and RR = 0.62 [95% CI 0.44–0.88], respectively, with no effect on neoplasm risk; however, serious infections significantly increased during anti-TNF treatment (RR = 1.48 [1.18–1.85]) compared to classical DMARD treatment [123]. Another meta-analysis of 28 studies of patients with RA reported that anti-TNF treatment was significantly associated with an overall CV risk reduction of approximately 30% [88]. Cheung et al. analyzed the effect of anti-TNF therapy on subclinical atherosclerosis, and six studies measured at least one parameter before and after treatment (24th and 52nd weeks): intima–media thickness, pulse wave velocity, and augmentation index; they found that anti-TNF therapy had no effect on all three parameters at the 24th week and on intima–media thickness at the 52nd week [124]. An older meta-analysis of 32 studies by Daien et al. reported increased total cholesterol and HDL levels and unchanged LDL levels with long-term anti-TNF therapy, with uncertain effects on CV risk [125]; however, the total cholesterol/HDL ratio was not significantly altered by anti-TNF therapy [74]. These anti-TNF therapeutic effects may reflect the normalization of lipid levels to those prior to RA due to suppression of inflammation [74,114]. A meta-analysis by Zhao et al., which included 6321 patients with RA from 11 randomized clinical trials, reported strong evidence of an increased incidence of hypertension associated with some anti-TNF therapies (OR = 1.8896, 95% CI: 1.35–2.65) as well as an increasing incidence of hypertension with longer therapy duration, especially for certolizumab but not for etanercept, tofacitinib, infliximab, and golimumab [126]. Results from a new Korean observational study with 996 patients with RA did not find an increased incidence of HA with biologics compared with classical DMARDs in general, but MTX had a lower incidence of hypertension, which could be explained by the hypothesis that MTX restores vasodilation-related adenosine levels in the body [127]. A neutral effect of anti-TNF agents on HA incidence was reported by Desai et al. from a large cohort study with 4822 patients using TNF-α inhibitors and 2400 using classical DMARDs [128]. Despite some methodological limitations, these studies strongly support the increased beneficial effects of anti-TNF agents compared to classical RA medications, probably due to more efficient inflammation control and successful achievement of long-term RA remission.

The effect of non-anti-TNF agents—abatacept, tocilizumab, sarilumab, anakinra, and rituximab—on CV risk has also been evaluated. Abatacept was found to have 20% greater CV risk reduction than anti-TNF agents; tocilizumab had the same effect on CV risk as MTX monotherapy; anakinra showed improved vascular and left ventricular function in a small placebo-controlled study; sarilumab and rituximab had a neutral effect on CV events; and canakinumab, an IL-1 inhibitor of 150 mg administered every 3 months, was associated with a significantly lower rate of myocardial infarction than the placebo [76,129,130,131,132,133,134]. Several studies have consistently reported that tocilizumab was associated with increased total cholesterol, HDL, LDL, and triglyceride levels [74,91]. The MEASURE study found that tocilizumab + MTX treatment did not increase the concentration of proatherogenic small and dense LDL particles, while antiatherogenic small and medium HDL particles were increased and structurally altered to a less inflammatory state than with MTX alone [135]. There have been no reports of an increase in CV events with tocilizumab [91]. These medications, especially abatacept and anakinra, are also strongly recommended for CVD prevention in RA.

#### 4.2.5. Small Molecule Inhibitors of JAK

Tofacitinib and baricitinib, newer small-molecule inhibitors of JAK, increase total and LDL cholesterol levels by 10–20% [136]. For baricitinib, a meta-analysis of six studies with 3552 patients reported significantly increased dose-dependent LDL and HDL with baricitinib use; the mean change was 13.15 mg/dL (95% CI: 8.89–17.42) and 5.40 mg/dL (95% CI: 3.07–7.74), respectively. Although the increased relative risk of CV events was not statistically significant, an association may exist [130]. A recently published pooled cohort study of 3492 patients with RA, with more than 7860 patient-years of exposure to baricitinib did not reveal a significant association between baricitinib treatment and the occurrence of CV events or congestive heart failure [137]. The influence of biologic agents and tofacitinib, a JAK inhibitor, on the lipid profile of patients with RA was analyzed in a meta-analysis of 20 articles by Soto et al.; they reported increased cholesterol (OR 4.64; CI 95% 2.71, 7.95, *p* < 0.001), HDL (OR 2.25; 95% CI 1.14, 4.44, *p* = 0.020), and LDL (OR 4.80; 95% CI 3.27, 7.05, *p* < 0.001) levels, but despite this effect, better inflammation control with those medications appears to result in lower mortality and incidence of cardiovascular events. However, other biologic or non-biologic agents were not included in the analysis [138].

Other than the ENTRACTE trial, which compared tocilizumab and etanercept, no head-to-head study has been conducted on the impact of biological agents on CV risk and safety. The ENTRACTE trial reported no significant difference in CV events between the tocilizumab and etanercept groups [85]. Some observational studies have reported a higher incidence of myocardial infarction among older patients with anti-TNF inhibitors than with abatacept and tocilizumab and no difference in CV risk when comparing tocilizumab with abatacept [76,139].

#### 4.2.6. Statins role in CVD prevention

The use of statins in the management of CVDs seems to have a neutral effect on RA development, and the risk of RA may be lower in patients with a longer duration or greater intensity of statin use [139]. Treatment with statins is beneficial in lowering CV risk in patients with RA due to their lipid-lowering and other pleiotropic effects that slow down coronary non-calcified plaque progression and suppress the effects of inflammation on plaque progression and CAC [77,118,141,142]. A meta-analysis of 11 relevant studies reported a standardized mean difference in DAS28 of −0.55 (95% CI: −0.83 to −0.26, *p* = 0.0002), supporting the positive effect of statins on RA [143]. However, the indiscriminate use of statins is not recommended in all patients with RA, and CV risk assessment and appropriate statin use according to the guidelines for primary and secondary CVD prevention in this population is necessary [17].

### 4.3. The Role of Inflammatory Markers in RA Activity and CV Risk Assessment

Biomarkers are used in disease diagnosis, treatment, and monitoring of disease progression and complications. They are also used to determine regression or remission of the disease. Although they are not always sufficiently sensitive and specific in certain situations, they still serve as a useful tool for monitoring the disease. Notably, RA activity reflects CV risk.

CRP is one of the most used markers worldwide and is routinely evaluated as a marker of systemic inflammation in RA [144]. Due to its limited specificity, CRP is most often utilized in conjunction with another blood biomarker. A low level of this biomarker indicates disease stability and the effectiveness of therapy. When using highly specific therapy directed against precisely defined inflammatory cytokines, monitoring the serum levels can facilitate the assessment of the treatment success and disease stability. In the early stages of inflammation, IL-6 is a key proinflammatory factor that causes a variety of cells to produce and secrete acute-phase proteins. Infection-related neutrophil generation and activation, B-cell proliferation and differentiation, immunoglobulin synthesis, and T-cell proliferation and differentiation are all stimulated by IL-6. The onset of inflammation and the change from acute to chronic inflammation are both significantly influenced by IL-6 levels [145]. Serum IL-6 levels stand as a reflective biomarker of RA disease activity [146].

TNF- is crucial to understanding the pathogenesis of RA. The expression of serum TNF- may make early RA more inflammatory [147]. As a result, it is necessary to test patients with RA for this cytokine to keep track of disease activity, which may be helpful for patients who are undergoing anti-TNF therapy. The inflammatory response is further enhanced by TNF-α, which is a potent inducer of other proinflammatory cytokines and chemokines [148]. Rheumatoid factors and anti-CCP, which are diagnostic markers of RA, can be used to track the progression of the disease.

## 5. Conclusions

In conclusion, to prevent CV disease in patients with RA, two main complementary strategies were considered. They were strict inflammation control with as few flares as possible and the management of modifiable risk factors.

Since CV morbidity and mortality in RA are alarmingly high, it is crucial to comprehend the mechanisms that cause and control atherosclerosis so that highly specific treatment plans can be created to reduce the CV health burden that patients with RA bear. The development of atherogenic foam cells is aided by proinflammatory cytokines, including IL-6 and TNF-α. The creation of a proatherogenic environment favors the development of atherosclerotic diseases because of endothelial dysfunction and RA-derived autoantibodies, which increase the inflammatory potential of macrophages. Understanding the potential interactions between inflammation and traditional cardiovascular (CV) risk factors in driving atherosclerosis in rheumatoid arthritis (RA) is a crucial area of investigation that requires further exploration.

Achieving and maintaining long-term RA remission using novel therapeutic agents is crucial. Early recognition and strict control of modifiable risk factors based on these guidelines are paramount. Effective patient education, implementation of these measures, increased surveillance, early active identification of risk factors by general practitioners and specialists, an interdisciplinary approach, and accessibility of the health care system play key roles in achieving these goals.

## Data Availability

The list and data from all used articles in this review can be provided upon request.

## Supplementary Materials

The following supporting information can be downloaded at https://www.mdpi.com/article/10.3390/medicina59091550/s1, Table S1: articles characteristics and comments included in review.
