# Supplementary material for: Role of Inflammatory Cytokines in Rheumatoid Arthritis and Development of Atherosclerosis: A Review
Source: Medicina (Kaunas). 2023 Aug 26;59(9):1550. doi: 10.3390/medicina59091550 (PMC10534747; doi:10.3390/medicina59091550)
Supplement: Supplementary file 1 [file medicina-59-01550-s001.zip › medicina-2562302-supplementary.pdf]

Table S1. articles characteristics and comments included in review.

FIRST AUTHOR	STUDY TYPE	YEAR OF PUBLICATI-ON	MAYOR AREA IN ARTICLE	TOPIC	NUMBER OF STUDIES AND PARTICIPANTS (CUMULATIVE FOR REVIEWS AND MEHTA-ANALYSES)	MAYOR LIMITATIONS AND OBSERVATION
Mitchell, D. M.[1]	PROSPECTIVE LONGITUDINAL STUDY	1986.	GENERAL	MORTALITY	805 PARTICIPANTS	
Scott, D. L.[2]	PROSPECTIVE LONGITUDINAL STUDY	1987.	GENERAL	MORTALITY	112 PARTICIPANTS	ONE CENTRE STUDY, OLD THERAPY
Arnett, F. C. [3]	COMPARATIVE STUDY	1988.	GENERAL	REVISED RA CLASSIFICATION 1987 CRITERIA	524 PARTICIPANTS	
Aletaha, D.[4]	SYSTEMATIC REVIEW OF COHORT STUDIES	2010.	GENERAL	EULAR 2010 RA CLASIFFICION CRITERIA	3115 PARTICIPANTS	
Lee, D. M. [5]	REVIEW	2001.	GENERAL	PATOPHISIOLOGY, THERAPY	93 STUDIES	
Symmons, D. P. M. [6]	REVIEW	2002.	GENERAL	EPIDEMIOLOGY	78 STUDIES	
Sakai, R. [7]	POPULATION BASED CROSS SECTIONAL STUDY	2016.	GENERAL	CVD PREVALENCE	2762 RA AND 27620 NON-RA PARTICIPANTS	
Koivuniemi, R. [8]	PROSPECTIVE LONGITUDINAL STUDY	2009.	GENERAL	AUTOPSY STUDY OF CVD	960 PARTICIPANTS	ONE CENTRE STUDY, OLD THERAPY
Maradit-Kremers, H. [9]	POPULATION BASED COHORT STUDY	2005.	GENERAL	CVD, CV RISK	603 PARTICIPANTS	
Sokka, T.[10]	REVIEW	2009.	GENERAL	MORTALITY OF CVD	124 STUDIES, > 100000 P	
Meune, C.[11]	MEHTA-ANALYSIS	2010.	GENERAL	MORTALITY OF AIM, ICV	17 STUDIES, > 124894 PARTICIPANTS	SIGNIFICANT HETEROGENEITY FOR AIM AND STROKE REPORTS, STANDARDIZED MORTALITY RATIO AND INCIDENCE RATE RATIO NOT ADJUSTED FOR CV RISK FACTORS
Meune, C. [12]	MEHTA-ANALYSIS	2009.	GENERAL	CVD, CV RISK	17 STUDIES, > 91916 PARTICIPANTS	SIGNIFICANT HETEROGENEITY AMONG STUDIES,
Avina-Zubieta, J. A. [13]	MEHTA-ANALYSIS	2008.	GENERAL	CV RISK	24 STUDIES, >111758 PARTICIPANTS	SIGNIFICANT HETEROGENEITY AMONG STUDIES, ONE SUBGROUP DID NOT SHOW SIGNIFICANTLY INCREASED RISK FOR CVD

Meyer, P. W. A. [14]	REVIEW	2018.	GENERAL , CHRONIC INFLAMMATION	INFLAMMATION MECHANISMS, CV RISK, TEHRAPY	55 STUDIES WITH LARGE NUMBER OF PARTICIPANTS	NARRATIVE REVIEW
Siebert, S.[15]	POPULATION BASED CROSS SECTIONAL STUDY	2016.	GENERAL	CVD AND DIABERES PREVALENCE	> 502649 PARTICIPANTS	CROSS SECTIONAL STUDY, SELF REPORT ON DIAGNOSIS, THERAPY,
Van Doornum, S.[16]	REVIEW	2002.	GENERAL	MORTALITY, CV RISK, INFLAMMATION	30 STUDIES, > 200000 PARTICIPANTS	4 HAD STUDIES NO INCREASED MORTALITY, INACCURATE DEATH CERTIFICATE SUSPECTED, STUDY HETEROGENEITY
Visseren, F.L.[17]	MEHTA-ANALYSIS, GUIDELINES	2021.	TRADITIONAL RISK FACTORS	GUIDELINES	> 800 STUDIES ANALYZED BY TASK FORCE	
Panoulas VF. [18]	REVIEW	2008.	TRADITIONAL RISK FACTORS	HA PREVALENCE	31 STUDIES, > 30 MILLION PARTICIPANTS	
Innala L.[19]	PROSPECTIVE CLINICAL STUDY	2016.	TRADITIONAL RISK FACTORS	RA COMORBIDITIES, INFLAMMATION	950 PARTICIPANTS	
Erba G[20]	PROSPECTIVE STUDY	2015.	TRADITIONAL RISK FACTORS	CVD, CV RISK SCORES	198 PARTICIPANTS	
Gherghe AM. [21]	COMPARATION OF CROSS SECTIONAL STUDIES	2015.	TRADITIONAL RISK FACTORS	HA PREVALENCE	1334 PARTICIPANTS	SELF REPORTING COMORBIDITIES (There is a trend for decrease in the prevalence of CVD in the French population. Data from DESIR were collected during the same period as the general population data, but the ESPOIR data were gathered prior to that date, which might have an influence when comparing comorbidities)
Boyer JF. [22]	MEHTA-ANALYSIS	2011.	TRADITIONAL RISK FACTORS	CV RISK PREVALENCE	6669 PARTICIPANTS	SIGNIFICANT HETEROGENEITY AMONG STUDIES FOR DIABETES AND HDL.
Heliovaara, M. [23]	PROSPECTIVE STUDY	1993.	TRADITIONAL RISK FACTORS	SMOKING	52818 PARTICIPANTS	
La Hoz, J.C.-D. [24]	MEHTA-ANALYSIS	2013.	TRADITIONAL RISK FACTORS	SMOKING	27 STUDIES	

Klareskog, L. [25]	POPULATION BASED CASE CONTROL STUDY	2006.	TRADITION AL RISK FACTORS	SMOKING, GENOTYPE	> 90000 PARTICIPANTS	CASE CONTROL STUDY
Welsing, P. M. J. [26]	PROSPECTIVE STUDY	2001.	TRADITION AL RISK FACTORS	FUNCTIONAL CAPACITY	378 PARTICIPANTS	JOINT DESTRUCTION AND FUNCTION CAPACITY WAS NOT ALWAYS DETERMINATE AT SAME TIME, 1.5 MONTHS TO 3 YEARS DERIVATION WERE ACCEPTABLE
Piepoli, M. F. [27]	MEHTA-ANALYSIS, GUIDELINES	2016.	TRADITION AL RISK FACTORS	GUIDELINES 2016 VERSION	> 570 STUDIES ANALYZED BY TASK FORCE	UPDATED IN 2021
Baghdadi LR. [28]	MEHTA-ANALYSIS	2015.	TRADITION AL RISK FACTORS	CV RISK	10 STUDIES, > 4388 PARTICIPANTS	ONE STUDY WAS CASE-CONTROL, SEVEN RETROSPECTIV E COHORTS, ONE WAS CROSS SECTIONAL
Jiang, P. [29]	MEHTA-ANALYSIS	2015.	TRADITION AL RISK FACTORS	DIABETES	19 STUDIES, > 1.3 MILLION PARTICIPANTS	11 CASE CONTROL STUDIES
Guin, A.[30]	PROSPECTIVE STUDY	2019.	TRADITION AL RISK FACTORS	INFLAMMTION, ATHEOSCLEROS IS, DMARDs	83 PARTICIPANTS	LIKELIHOOD OF LONG DURATION RA PATIENTS HAVE HIGHER HOMA-IR IS QUITE HIGH BUT STUDY SAMPLE IS SMALL.
Dougados, M.[31]	INTERNATION AL CROSS SECTIONAL STUDY	2020.	TRADITION AL RISK FACTORS	CVD, CV RISK	3920 PARTICIPANTS	NOT ALL COMORBIDITIE S INCLUDES SUCH TUBERCULOSIS , QUESTIONABL E ENROLMENT AND DISEASE ACTIVITY ASSESSMENT, DIVERSE IN QUESTIONS INTERPRETATI ON IN FORMS, DIFFERENCES IN DEPRESSION PREVALENCE.
Stavropoul os-Kalinoglou, A. [32]	CROSS SECTION STUDY	2007.	TRADITION AL RISK FACTORS	BMI	641 PARTICIPANTS	CROSS SECTION STUDY ON ASIAN POPULATION
Castro, L.L.[33]	CROSS SECTION STUDY	2018.	TRADITION AL RISK FACTORS	CV RISK	133 PARTICIPANTS	CROSS SECTION STUDY

Beinsberger, J. [34]	REVIEW	2014.	TRADITIONAL RISK FACTORS AND CHRONIC INFLAMMATION	RA AGRAVATES CVD	55 STUDIES	
Nowak, B. [35]	PROSPECTIVE OBSERVATIONAL STUDY	2016.	TRADITIONAL RISK FACTORS AND CHRONIC INFLAMMATION	CV RISK, ESR, DURATON AND ACTIVITY OF RA, ANTI-CCP, LDL	61 PARTICIPANTS	
Giles, J. T.[36]	MEHTA-ANALYSIS	2011.	TRADITIONAL RISK FACTORS AND CHRONIC INFLAMMATION	LDL	6035 PARTICIPANTS	ONLY 4 COHORT STUDIES WITH DIFFERENCES IN INCLUSION/EXCLUSION CRITERIA, DANA COLLECTED, GEOGRAPHIC LOCATION, LIMITED STATISTICAL POWER IN SUBGROUP ANALYSES
McGrath, C. M. [37]	REVIEW	2015.	TRADITIONAL RISK FACTORS AND CHRONIC INFLAMMATION	LIPID METABOLISAM, LIPID PARADOX	100 A, > 9000 PARTICIPANTS	NUMBER ANT TYPE OF ANALYZED STUDIES MISSING
Gregersen, P. K.[38]	REVIEW	1987.	CHRONIC INFLAMMATION	GENETIC STUDY, CCP	STUDY ON ANIMALS	
McInnes, I. B. [39]	REVIEW	2011.	CHRONIC INFLAMMATION	SYNOVITIS IN RA, PATHOGENESIS OF ORGAN DAMAGE IN RA	174 STUDIES	
Wang, D. [40]	REVIEW	2019.	CHRONIC INFLAMMATION	LUNG DISEASE IN RA	169 STUDIES	
Londei, M. [41]	CASE REPORT	1987.	CHRONIC INFLAMMATION	INFLAMMATION MECHANISMS - GENETIC ANALYZE	1 CASE REPORT	CASE REPORT
Glant, T. T. [42]	PROSPECTIVE STUDY	2011.	CHRONIC INFLAMMATION	INFLAMMATION MECHANISMS	STUDY ON ANIMALS	
Verheijden, G. F. M.[43]	PROSPECTIVE STUDY	2011.	CHRONIC INFLAMMATION	INFLAMMATION MECHANISMS, DETECTING RF AND ANTI-CCP ON ANIMAL MODELS	STUDY ON ANIMALS	
Smolen, J. S.[44]	REVIEW	2018.	CHRONIC INFLAMMATION	INFLAMMATION MECHANISMS, EPIDEMIOLOGY, DIAGNOSIS, TREATHMENT OF RA	261 STUDIES	

Burmester, G. R. [45]	REVIEW	2014.	CHRONIC INFLAMMATION	INFLAMMATION MECHANISMS, CYTOCINE DIRECTED THERAPIES	134 STUDIES	
Carbone, F.[46]	REVIEW	2020.	CHRONIC INFLAMMATION	INFLAMMATION MECHANISMS, ATHEOSCLEROSIS, CVD	204 STUDIES	
Mewar, D.[47]	CROSS SECTIONAL STUDY	2006.	CHRONIC INFLAMMATION	INFLAMMATION MECHANISMS - RADIOGRAPHIC STUDY, ANTI-CCP, RF	872 PARTICIPANTS	POTENTIAL BIAS WITH PATIENTS WITH MILD DISEASE
Sokolove, J.[48]	PROSPECTIVE STUDY	2012.	CHRONIC INFLAMMATION	INFLAMMATION MECHANISMS, ANTI-CCP	162 PARTICIPANTS	LABORATORY METHODOLOGY LIMITATIONS WITH SERONEGATIVE RA, OVERESTIMATE ANTI CCP RESULTS IN YOUNGER POPULATION
Lopez-Longo, F. J.[49]	RETROSPECTIVE COHORT STUDY	2009.	CHRONIC INFLAMMATION	INFLAMMATION MECHANISMS, ANTI-CCP, CVD	937 PARTICIPANTS	ANTI-CCP HAS LOW FREQUENCY IN ISCHEMIC CVD AND HIGH IN RA
Sokolove, J.[50]	CROSS SECTION STUDY	2013.	CHRONIC INFLAMMATION	CHRONIC INFLAMMATION - ANTI CCP, ATHEOSCLEROSIS	134 PARTICIPANTS	INABILITY TO DEMONSTRATE DIRECT ANTI-CCP COMPLEX IN PLAQUES DO TO UNAVAILABILITY OF RAPID AUTOPSY TISSUE SPECIMENS
Geraldino-Pardilla, L.[51]	CROSS SECTION STUDY	2018.	CHRONIC INFLAMMATION	INFLAMMATION MECHANISMS, FDG UPTAKE INFLAMMATION DETECTION, CV RISK	91 PARTICIPANTS	CROSS SECTION STUDY, FALSE POSITIVE FDG UPTAKE
DeMizio, D. J. [52]	REVIEW	2020.	CHRONIC INFLAMMATION AND MEDICATIONS	INFLAMMATION MECHANISMS AND MEDICATIONS - CV DEATH, CV RISK, INFLAMMTION MARKERS, ATHEROSCLEROSIS	90 STUDIES, > 100000 PARTICIPANTS	
Liang, K. P. [53]	POPULATION BASED PROSPECTIVE STUDY	2009.	CHRONIC INFLAMMATION	CVD, MORTALITY, ANTI CCP, ANA	14934 PARTICIPANTS	LACK OF CRP VALUES, SMALL NUMBER OF ANTI-CP POSITIVE PARTICIPANTS, LACK OF VALIDATION OF CONFOUNDING

						, COMORBIDITIES, RA AND CVD ASSESSMENTS ACCORDING TO ESTABLISHED CRITERIA
Humphreys, J. H., [54]	LARGE CLINICAL TRIAL	2014.	CHRONIC INFLAMMATION	MORTALITY, ANTI-CCP, RF	4962 PARTICIPANTS	NO POOL ANALYZES FROM TWO COHORTS, STUDY DOES NOT ACCOUNT ALL PREDICTIONS OF MORTALITY IN RA, ASSUMPTION THAT ANTIBODY STATUS IS NOT FIXED IN RA.
McCoy, S. S.[55]	POPULATION BASED PROSPECTIVE STUDY	2013.	CHRONIC INFLAMMATION	MYOCARDIAL INFARCTION	231 PARTICIPANTS	RETROSPECTIVE STUDY WITH DANA DEPENDENT ON DOCUMENTATION, NO DANA ABOUT DISEASE ACTIVITY IN TIME OF AIM, NO DANA ABOUT NSAID, CV RISK FACTORS, LIMITATIONS IN STUDY POWER IN RA SUBGROUP ANALYSIS
Mackey, R. H.[56]	PROSPECTIVE LONGITUDINAL STUDY	2015.	CHRONIC INFLAMMATION	INFLAMMATION MECHANISMS, CV RISK, MORTALITY	160000 PARTICIPANTS	PART OF RA WAS NOT DIAGNOSED BY CLINICAL EXAM, NO INFORMATION ON DISEASE DURATION, SOME ANTI-CCP RESULTS ARE QUESTIONABLE, POSSIBLE CONFOUNDING IN MEDICATION USE, SELF REPORTING ON CHOLESTEROL AND ANTILIPEMIC DRUGS USE.
Innala, L.[57]	LONG TERM PROSPECTIVE OBSERVATIONAL STUDY	2010.	CHRONIC INFLAMMATION	CVD, CV RISK, DMARD	442 PARTICIPANTS	RISK OF CONFOUNDING REGARDING MEDICATIONS EFFECTS

Pawlik A. [58]	CROSS SECTION STUDY	2003.	CHRONIC INFLAMMATION	INFLAMMATION MECHANISMS - CD4, CD 28 CELLS, CVD	42 PARTICIPANTS	CROSS SECTION STUDY
Winchester, R. [59]	PROSPECTIVE COHORT STUDY	2016.	CHRONIC INFLAMMATION	INFLAMMATION MECHANISMS - CORONARY ARTERY CALCIFICATIONS, MONONUCLEAR CELLS	73 PARTICIPANTS	STUDY DESIGN DOES NOT ALLOW TO DISTINGUISH BETWEEN TWO INTERPRETATION OF INFLAMMATORY ROLE IN ATHEROSCLEROSIS
Nakajima, T. [60]	PROSPECTIVE STUDY	2003.	CHRONIC INFLAMMATION	INFLAMMATION MECHANISMS IN ACS CD4+CD28null T CELLS HAVE UNIQUE GENE EXPRESSION PROFILE AVOIDING RECEPTOR ACTIVATION	73 PARTICIPANTS	
Dumitriu, I. E. [61]	PROSPECTIVE STUDY	2010.	CHRONIC INFLAMMATION	INFLAMMATION MECHANISMS IN ACS COSTIMOLATORY PATHWAYS ARE ALTERED IN CD4(+)CD28(null) T CELLS.	94 PARTICIPANTS	
Lopez-Mejias, R. [62]	REVIEW	2016.	CHRONIC INFLAMMATION	INFLAMMATION MECHANISMS, CV RISK	356 STUDIES	
Liuzzo, G.[63]	PROSPECTIVE STUDY	2005.	CHRONIC INFLAMMATION	INFLAMMATION MECHANISMS - ALTERATION OF T-CELL REPERTOIRE IS ASSOCOATIE WITH CVD	120 PARTICIPANTS	
Libby, P. [64]	REVIEW	2007.	CHRONIC INFLAMMATION	INFLAMMATION MECHANISMS, ENDOTHELIAL DYSFUNCTION, ATHEOSCLEROSIS	55 STUDIES	
Swerdlow, D. I. [65]	MEHTA-ANALYSIS	2012.	CHRONIC INFLAMMATION	GENETIC STUDY OF IL6R IN CVD	40 STUDIES, > 133449 PARTICIPANTS	
Kaptoge, S. [66]	MEHTA-ANALYSIS	2014.	CHRONIC INFLAMMATION	INFLAMMATION HYPOTESIS IN VASCULAR DISEASES	17000 PARTICIPANTS	TNF-α WERE MEASURED AT START IN CASE-COHORT STUDY OF 1514 PARTICIPANTS AND 833 INCIDENT CVD EVENTS IN POPULATION-BASED PROSPECTIVE COHORTS.

van den Oever, I. A. M.[67]	REVIEW	2014.	CHRONIC INFLAMMATION	THROMBOEMBOLIC RISK	31 STUDIES	
Small, H. Y.[68]	REVIEW	2018.	CHRONIC INFLAMMATION	HA, OXIDATIVE STRESS	202 STUDIES	
Peters, M. J. L.[69]	PROSPECTIVE STUDY	2009.	CHRONIC INFLAMMATION	THROMBOSIS IN RA	42 PARTICIPANTS	
Habets, K. L. L.[70]	PROSPECTIVE STUDY	2015.	CHRONIC INFLAMMATION	THROMBOSIS IN RA, ANTI-CCP	86 PARTICIPANTS	
Zhou, Z. W.[71]	MEHTA-ANALYSIS	2020.	CHRONIC INFLAMMATION	PLATLETS IN CHR. INFLAMMATION	34 STUDIES; 17 on RA, 12 on AS, 3 on PsA and 2 on both RA and AS	
Agca, R. [72]	LONG TERM PROSPECTIVE STUDY	2020.	CHRONIC INFLAMMATION	CV RISK, INFLAMMATION INDEPENDANT CONTRIBUTOR	2893 PARTICIPANTS	HOOM STUDY WAS 10 YEARS CAREE STUDY - POSSIBLE DIFFERENCES IN DEFINITIONS, DECLINE OF CVD IN PAST 10 YEARS - UNDERESTIMATION IN CV RISK, MTX AND BIOLOGICS ARE NOT REPRESENTATIVE AT STUDY BASSLINE - CHANGES IN GUIDELINES OVER TIME
Cugno, M. [73]	REVIEW	2010.	CHRONIC INFLAMMATION	COAGULATION ACTIVATION, INCREASED VASCULAR PERMEABILITY	48 STUDIES	
Choy, E. [74]	REVIEW	2014.	CHRONIC INFLAMMATION AND MEDICATIONS	INFLAMMATION MECHANISMS AND MEDICATIONS - CV RISK, INFLAMMTION CONTROL THERAPY	155 STUDIES	
Carbone, F.[46]	REVIEW	2020.	CHRONIC INFLAMMATION	CV RISK, CVD, ATHEOSCLEROSIS, INFLAMMATION MECHANISMS	204 STUDIES	
Myasoedova, E.[75]	RETROSPECTIVE MULTICENTER COHORT STUDY	2011.	CHRONIC INFLAMMATION	CVD, ESR, LIPID PARADOX	651 PARTICIPANTS	NO CAUSAL RELATIONSHIP CAN NOT BE MADE BETWEEN OUTCOMES AND INFLAMMATION AND LIPIDS, RETROSPECTIVE STUDY
Ridker, P. M. [76]	RANDOMIZED DOUBLE-BLIND	2017.	CHRONIC INFLAMMATION AND	THERAPY, LDL	10061 PARTICIPANTS	

	CLINICAL TRIAL		MEDICATIONS			
Goodson, N. J.[77]	PROSPECTIVE STUDY	2009.	CHRONIC INFLAMMATION AND MEDICATIONS	CVD, MORTALITY, NSAID	923 PARTICIPANTS	UNCLEAR IS THIS REPRESENTS UNMEASURED CONFOUNDERS INFLUENCING DECISION TO AVOID NSAID USE IN RA
Gonzalez-Gay, M. A.[78]	REVIEW	2005.	CHRONIC INFLAMMATION, TRADITIONAL CV RISK FACTORS	TRADTIONAL CV RISK FAVTORS, INFLAMMTION CONTRIBUTRS SIGINIFICANTLY	106 STUDIES	
Kerola, A. M.[79]	PROSPECTIVE STUDY	2013.	CHRONIC INFLAMMATION	STANDARDISER RATE RATIO FOR CORONARY DISEASE AND HA IN EARLY RA AND SERONEGATIVE IS AUGMENTET	7209 PARTICIPANTS	
Södergren, A. [80]	PROSPECTIVE STUDY	2010.	CHRONIC INFLAMMATION	IMT INCREASES WITH RA DURATION	79 PARTICIPANTS	LARGER RA GROUP, USE OF MEDICATIONS
Maga M.[82]	CASE REPORT	2021	CHRONIC INFLAMMATION	PSORIASIS AS A CAUSE OF CRITICAL LIMB ISCHEMIA WIN 27 YEAR OLD PATIENT WITHOUT CV RUSK FACTORS	1	CASE REPORT, SUCCESS OF INFLAMMATION CONTROL AND ARTICULAR INVOLVEMENT WERE NOT DESCRIBED
González-Gay, MA. [81]	REVIEW	2012.	CHRONIC INFLAMMATION	ENDOTHELIAL DYSFUNCTION	10 STUDIES	
Di Minno, MN. [83]	MEHTA-ANALYSIS	2015.	CHRONIC INFLAMMATION	ENDOTHELIAL DYSFUNCTION	20 STUDIES, 1688 PARTICIPANTS	
Ambrosino, P.[84]	MEHTA-ANALYSIS	2015.	CHRONIC INFLAMMATION	MORE ACTIVE INFLAMMATION INCREASES AORTIC AND ARTERIAL STIFFNES RVRN IN EARLY STAGE.	25 STUDIES, 3055 PARTICIPANTS	CASE CONTROL STUDIES WITH HETEROGENEITY, DIFFERENT INCLUSION/EXCLUSION CRITERIA, DIFFERENT RA EARLY DIAGNOSIS DEFINITION AND ACTIVITY, MANY CV RISK FACTORS WHICH INFLUENCE STIFFNESS MEASUREMENTS, DIFFERENCES AMONG DEVICES AND TECHNIQUES

						USED IN STUDIES
Giles, J. T.[88]	MEHTA-ANALYSIS	2019.	CHRONIC INFLAMMATION	LIPID PARADOX	4 STUDIES, 5825 PARTICIPANTS	ONLY 4 COHORT STUDIES WITH DIFFERENCES IN INCLUSION/EXCLUSION CRITERIA, DANA COLLECTED, GEOGRAPHIC LOCATION, LIMITED STATISTICAL POWER IN SUBGROUP ANALYSES
Paccou, J. [85]	CROSS SECTION COMPARATIVE STUDY	2014.	CHRONIC INFLAMMATION	CORONARY AND AORTIC CALCIFICATIONS ARE MORE PREVALENT AND SEVERE IN RA, MTX CAN REDUCE IT, EROSIVE ARTITIIS IS MAJOR DETERMINANT OF AORTIC CCALCIFICCATIONS.	150 P	CROSS SECTIONAL STUDY, INFLUENCE OF MTX DOSAGE AND DURATION WAS NOT VALIDATED, PROGNOSTIC SIGNIFICANCE OF CALCIFICATION PRESENCE, DIFFICULTY IN ESTIMATION OF CALCIFICATIONS IN ARTERIAL WALL BY MSCT, NON-CALCIFIED PLAQUES COULD NOT BE DIAGNOSED
McGettigan, P. [86]	REVIEW	2006.	MEDICATIONS	COX 2 INHIBITORS - ROFECOXIB AND CELCOXIB MAY NOT INCREASE CV RISK	23 S	CASE-CONTROL OR COHORT DESIGN
Kearney, P. M. [87]	MEHTA-ANALYSIS	2006.	MEDICATIONS	COX 2 INHIBITORS - SELECTIVE COX 2 INHIBITORS INCREASE CV RISK.	138 STUDIES, 145373 PARTICIPANTS	SMALL NUMBER OF ADVERSE EVENTS, LIMITED DANA ON CV RISK AND GASTROINTESTINAL EFFECTS

Roubille, C. [88]	MEHTA-ANALYSIS	2015.	MEDICATIONS	TNF ALPHA INH, MTX, NSAID, GLUCOCORTICOIDES	34 STUDIES (28 RA,6 PSA), 456734 PARTICIPANTS	POSSIBLE UNDERPOWERED FOR MTX CV RISK LOWERING DETECTION
Westlake, S. L.[106]	MEHTA-ANALYSIS	2011.	MEDICATIONS	TNF ALPHA INH, MTX	20 STUDIES, 1688 PARTICIPANTS	OBSERVATIONAL STUDIES, 2 STUDIES HAVE SIGNIFICANT BIAS, CONFOUNDING factors: HIGH RA ACTIVITY HAS HIGH CV RISK, MTX USE, ADJUSTMENT OF BIOLOGICS TO RA ACTIVITY
Cabassi, A.[89]	REVIEW	2020.	MEDICATIONS	NSAID	111 STUDIES, > 7 MILLION PARTICIPANTS	
Gargiulo, G. [90]	REVIEW	2014.	MEDICATIONS	NSAID	90 STUDIES, > 100000 PARTICIPANTS	
Gasparyan, A. Y. [91]	REVIEW	2012.	MEDICATIONS	MTX, HYDROXYCHLOROQUINE	240 STUDIES	
Szeto, C. C. [92]	MEHTA-ANALYSIS	2020.	MEDICATIONS	NSAID	329 STUDIES	
Zheng, L. Y. [93]	REVIEW	2014.	MEDICATIONS	NSAID	23 STUDIES	
Caldwell, B. [94]	MEHTA-ANALYSIS	2006.	MEDICATIONS	COX 2 INHIBITORS	PRIMARY 4 STUDIES WITH 4422 PARTICIPANTS , SECONDARY 6 STUDIES WITH 12780 PARTICIPANTS	FOR CELECOXIB ALL STUDIES WERE UNDERPOWERED TO DETECT CVD, PROBLEMS WITH REPORTING SIDE-EFFECTS AND ENDPOINTS.
Helin-Salmivaara, A.[95]	LARGE CASE CONTROL STUDY	2006.	MEDICATIONS	NSAID AND COX2 INHIBITORS	172258 PARTICIPANTS	
Schneeweiss, S. [96]	COMPARATIVE STUDY	2006.	MEDICATIONS	NSAID AND COX2 INHIBITORS	49711 PARTICIPANTS	
Fabule, J. [97]	COMPARATIVE STUDY	2014.	MEDICATIONS	NSAID AND COX2 INHIBITORS	19 STUDIES, > 22.5 MILLION PARTICIPANTS	LOW OUTCOMES NUMBER, NOT ALL NSAIDs INCLUDED, NO CARDIOVASCULAR SIDE EFFECTS DETECTION OR ANALYSES
del Rincon, I.[98]	RANDOMIZED CONTROLLED TRIAL	2014.	MEDICATIONS	GLUCOCORTICOIDES	779 PARTICIPANTS	
Soubrier, M. [99]	REVIEW	2014.	MEDICATIONS	GLUCOCORTICOIDES, NSAID, MTX, THF ALPHA INH	59 STUDIES	

Ravindran, V.[100]	MEHTA-ANALYSIS	2009.	MEDICATIONS	GLUCOCORTICOIDES	6 STUDIES, 689 PARTICIPANTS	REDUCED POSSIBILITY TO DETECT RARE ADVERSE EFFECTS
Ruyssen-Witrand, A.[101]	REVIEW	2011.	MEDICATIONS	GLUCOCORTICOIDES	37 STUDIES	3 ABSTRACTS
Agca, R. and EULAR experts [102]	MEHTA-ANALYSIS	2017.	MEDICATIONS	GUIDELINES	234 RA, 17 AS, 13 PsA STUDIES	
Suissa, S.[103]	CASE CONTROL STUDY	2006.	MEDICATIONS	DMARD (MTX), GLUCOCORTICOIDES, NSAID, COX2 INH.	107908 PARTICIPANTS	CASE CONTROL STUDY, NO AIM OUTCOME ANALYZED, POSSIBLE CONFOUNDING FOR DMARD PRESCRIBING
Rempenault, C.[104]	MEHTA-ANALYSIS	2018.	MEDICATIONS	HYDROXYCHLOROQUINE	16 STUDIES	DANA EXTRACTED BY ONE INVESTIGATOR AND CHECKED BY ANOTHER ONE.
Widdifield, J.[105]	PROSPECTIVE COHORT STUDY	2019.	MEDICATIONS	MTX	23994 PARTICIPANTS	CONFOUNDING WITH MTX DOSAGE AND PRESENCE OF COMORBIDITIES, NO DANA ABOUT OTHER MEDICATIONS WITH POSSIBLE CONFOUNDING EFFECT OR CV RISK FACTORS
Micha, R. [107]	MEHTA-ANALYSIS	2011.	MEDICATIONS	MTX	10 STUDIES	OBSERVATIONAL STUDIES - BIAS IS POTENTIALLY EVIDENT(funnel plot, Begg's test, p = 0.06); EXCLUDING STUDIES WITH EXTREME RISK DID NOR ALTER RESULTS (relative risk 0.81, 95% CI 0.74 to 0.89).
Morris, S. J.[108]	COMPARATIVE STUDY	2011.	MEDICATIONS	HYDROXYCHLOROQUINE	706 PARTICIPANTS	CONFOUNDERS THAT AFFECT LIPID PROFILE WERE NOR AVAILABLE: FAMILY HISTORY, CENTRAL ADIPOSITY, PHYSICAL ACTIVITY, NO

						DIRECT RA ACTIVITY MEASURED
Li, H. Z. [109]	REVIEW OF MEHTA-ANALYSIS	2019.	MEDICATIONS	HYDROXYCHLOROQUINE	6 STUDIES, 689 PARTICIPANTS	METHODOLOGY QUESTIONABLE TO Rempenault, C. MEHTA ANALYSIS
Charles-Schoeman, C. [110]	RANDOMIZED CLINICAL TRIAL	2016.	MEDICATIONS	MTX, SSZ, HYDROXYCHLOROQUINE	416 PARTICIPANTS	STUDY INCLUDES EARLY RA WITH HIGH DISEASE ACTIVITY, NAIVE TO DMARDs, ALL SEROPOSITIVE, LIMITED DANA ON GLUCOCORTICOIDS AND STATINS, NO DANA ON CARDIOVASCULAR EXERCISE
Solomon, D. H. [111]	CASE CONTROL STUDY	2006.	MEDICATIONS	MTX, BIOLOGICS, GLUCOCORTICOIDES, AZATHIOPRINE, CYCLOSPORINE, LEFLUNOMIDE	3501 PARTICIPANTS	CASE CONTROL STUDY, CONFIDENCE LIMITS ABOUT CV RISK ESTIMATION, POSSIBLE MISCLASSIFICATION OF ELIGIBLE PATIENTS OR CVD, MISDIAGNOSIS OF RA
Smolen, J. S. and EULAR TASK experts 2013. Update[112]	MEHTA-ANALYSIS	2014.	MEDICATIONS	GUIDELINES		
Solomon, D. H. [113]	COMPARATIVE STUDY	2013.	MEDICATIONS	TNF ALPHA INH, DMARDs	20243 PARTICIPANTS	MISCLASSIFICATION IN ENDPOINT AND EXPOSURE (RA AND CVD), SURVEILLANCE BIAS
Toussirot, E. [114]	EXPERT OPINION OD DRUG SAFETY	2015.	MEDICATIONS	TNF ALPHA INH	EXPERT OPINION	EXPERT OPINION

Barnabe, C. [115]	MEHTA-ANALYSIS	2011.	MEDICATIONS	TNF ALPHA INH	16 STUDIES, 108328 PARTICIPANTS	HETEROGENEITY AMONG COHORT STUDIES, POSSIBLE PUBLICATION BIAS. EFFECT FROM RCT IS UNDERPOWERED WITH WIDE 95% Cls AND CVD WERE SECONDARY OUTCOMES, RCT HAVE TREAD TOWARDS DECREASED RISK
Jacobsson, L. T. H. [116]	PROSPECTIVE COHORT STUDY	2005.	MEDICATIONS	TNF ALPHA INH	983 PARTICIPANTS	
Ljung, L.[117]	PROSPECTIVE STUDY	2016.	MEDICATIONS	TNF ALPHA INH	41093 PARTICIPANTS	
Karpouzas, G. A. [118]	PROSPECTIVE STUDY	2020.	CHRONIC INFLAMMATION AND MEDICATIONS	BIOLOGICS	100 PARTICIPANTS	
Singh, S. [119]	MEHTA-ANALYSIS	2020.	MEDICATIONS	TNF ALPHA INH, NON-TNF ALPHA INH, DMARDs (TOCILIZUMAB)	26 STUDIES, > 220000 PARTICIPANTS	OBSERVATIONAL STUDIES, ONE RANDOMIZED CLINICAL TRIAL COMPARED CV SAFETY OF TOCILIZUMAB AND ETANERCEPT, POSSIBLE CONFOUNDING REGARDING MEDICATIONS FOR RA AND COMORBIDITIES PRESCRIBING, POSSIBLE PUBLICATION BIAS, UNCERTAIN RA DURATION
Chung, E. S.[120]	PROSPECTIVE CLINICAL TRIAL	2003.	MEDICATIONS	INFLIXIMAB	150 PARTICIPANTS	
Leporini, C.[121]	MEHTA-ANALYSIS	2018.	MEDICATIONS	TNF ALPHA INH	12 STUDIES	
Castagne, B.[122]	MEHTA-ANALYSIS	2019.	MEDICATIONS	TOCILIZUMAB	19 STUDIES, > 110000 PARTICIPANTS	HETEROGENEITY FOR MACE OUTCOMES, NO DATA FOR BASELINE CV RISK, TRANSIVITY HYPOTHESIS HAS NOT BE TESTED

Divonne, M. D. [123]	MEHTA-ANALYSIS	2017.	MEDICATIONS	TNF ALPHA INH	27 STUDIES	
Cheung, T. T. [124]	MEHTA-ANALYSIS	2015.	MEDICATIONS	TNF ALPHA INH	20 STUDIES	
Daien, C. I. [125]	MEHTA-ANALYSIS	2012.	MEDICATIONS	TNF ALPHA INH	13 S	
Zhao, Q. W. [126]	MEHTA-ANALYSIS	2015.	MEDICATIONS	TNF ALPHA INH	11 STUDIES, 6321 PARTICIPANTS	STRICT INCLUSION AND EXCLUSION CRITERIA FOR ANALYZED TRIALS, POOLED DANA TENDS TO OVERESTIMATE OR UNDERESTIMATE TREATMENT EFFECT, HETEROGENEITY OF ANALYZED TRIALS.
Kim, S. K. [127]	LARGE PROSPECTIVE STUDY	2020.	MEDICATIONS	TOCILIZUMAB, ABATACEPT	996 PARTICIPANTS	POSSIBLE INACCURATE HA DIAGNOSIS, NO OTHER CV RISK FACTORS WERE ASSED
Desai, R. J.[128]	PROSPECTIVE STUDY	2016.	MEDICATIONS	TNF ALPHA INH, NONBIOLOGICS - MTX	7222 PARTICIPANTS	NO DATA ON RA ACTIVITY, POSSIBLE MISCLASSIFICATION BIAS ON DIAGNOSES OF RA AND COMORBIDITIES
Jin, Y. Z.[129]	PROSPECTIVE STUDY	2018.	MEDICATIONS	ABATACEPT	13036 PARTICIPANTS	OBSERVATIONAL STUDY, POSSIBLE CONFOUNDING BY PARTIALLY MEASURED OR UNMEASURED PARAMETERS, NO MEASURED DISEASE ACTIVITY, LIMITED STATISTICAL POWER IN SOME SECONDARY ANALYSES, POSSIBLE MISCLASSIFICATION IN OUTCOMES AND COMORBIDITIES, PROBLEMS WITH BASELINE RA SEVERITY AND DURATION AND CV RISK ESTIMATION

Schiff, M. H.[130]	CLINICAL TRIAL	2011.	MEDICATIONS	TOCILIZUMAB	8580 PARTICIPANTS	STRICT ENROLMENT CRITERIA AND MONITORING IN CLINICAL TRIAL SETTING
Fleischmann, R.[131]	CLINICAL TRIAL	2019.	MEDICATIONS	SARILUMAB	3509 PARTICIPANTS	
Ikonomidis, I. [132]	RANDOMIZED CONTROL TRIAL	2008.	MEDICATIONS	ANAKIRA	42 PARTICIPANTS	NO EXPLORATION OF CAUSALITY BETWEEN ANAKIRA VASCULAR AD LIVER FUNCTION, NO CORONARY DISEASE EXPLORED, NONINVASIVE ASSESSMENT OF VASCULAR AND LIVER FUNCTION, IMPROVED PHYSICAL ACTIVITY REDUCED DAS28 SCORE
van Vollenhoven, R. F. [133]	CLINICAL TRIAL	2013.	MEDICATIONS	RITUXIMAB	3194 PARTICIPANTS	DATA POOLED FROM DIVERSE STUDIES, WITH 2 STUDIES WERE PATIENTS RECEIVED ONE OR TWO RITUXIMAB DOSES, EXCLUDED SEVERE COMORBIDITIES AT BASELINE, SHORT OBSERVATION PERIOD FOR PLACBEO+MTX
Day, A. L. [134]	REVIEW	2019.	MEDICATIONS	DMARD	83 STUDIES	
McInnes, I. B. [134]	CLINICAL TRIAL	2015.	MEDICATIONS	TOCILIZUMAB	132 PARTICIPANTS	
Tanaka, Y.[136]	CLINICAL TRIAL	2011.	MEDICATIONS	MTX, TOFACITINIB	140 PARTICIPANTS	
Taylor, P. C. [137]	CLINICAL TRIAL	2019.	MEDICATIONS	BARICITINIB	3492 PARTICIPANTS	DVT/PE WERE NOT ASSES, NOT ALL ADVERSE EVENTS WERE DIAGNOSED BY IMAGING TOOLS, RESTRICTED DURATION MAY LIMIT DETECTION OF RARE ADVERSE EVENTS.

Souto, A. [138]	MEHTA-ANALYSIS	2015.	MEDICATIONS	TOCILIZUMAB, TOFACITINIB	25 STUDIES, > 4700 PARTICIPANTS	NO DATA FOR OTHER BIOLOGICS IN RA OR SpA
Zhang, J. [139]	RETROSPECTIVE COHORT STUDY	2016.	MEDICATIONS	ABATACEPT, ANTI TNF INH.	47193 PARTICIPANTS	RETROSPECTIVE STUDY
Myasoedova, E. [140]	MEHTA-ANALYSIS	2019.	MEDICATIONS	STATINS	8 STUDIES, > 1800000 PARTICIPANTS	5 COHORT, 3 CASE CONTROL
Soulaidopoulos, S. [141]	REVIEW	2018.	MEDICATIONS	STATINS	11 STUDIES	DIFFICULTY IN ASSESSING STATIN EFFECT O INFLAMMATION
Danninger, K. [142]	REVIEW	2014.	MEDICATIONS	STATINS	4896 PARTICIPANTS	
Xing, B. [143]	MEHTA-ANALYSIS	2015.	MEDICATIONS	STATINS	13 STUDIES , 737 PARTICIPANTS	2 STUDIES BASED ONLY ON CRP OR ESR, ALL WERE CONDUCTED IN ONE HOSPITAL WITH SMALL NUMBER OF PATIENTS, HETEROGENEITY IN STATIN TYPES, DOSAGE AND RA ACTIVITY ASSESSMENT
